# Advancement in COVID‐19 detection using nanomaterial‐based biosensors

**DOI:** 10.1002/EXP.20210232

**Published:** 2023-01-07

**Authors:** Phuoc Loc Truong, Yiming Yin, Daeho Lee, Seung Hwan Ko

**Affiliations:** ^1^ Laser and Thermal Engineering Lab Department of Mechanical Engineering Gachon University Seongnam Korea; ^2^ New Materials Institute Department of Mechanical Materials and Manufacturing Engineering University of Nottingham Ningbo China Ningbo China; ^3^ Applied Nano and Thermal Science Lab Department of Mechanical Engineering Seoul National University Gwanak‐gu Seoul Korea; ^4^ Institute of Advanced Machinery and Design (SNU‐IAMD)/Institute of Engineering Research Seoul National University Gwanak‐gu Seoul Korea

**Keywords:** colorimetric biosensor, COVID‐19 detection, electrochemical biosensor, field‐effect transistor biosensor, mass spectroscopy, plasmonic biosensor

## Abstract

Coronavirus disease 2019 (COVID‐19) pandemic has exemplified how viral growth and transmission are a significant threat to global biosecurity. The early detection and treatment of viral infections is the top priority to prevent fresh waves and control the pandemic. Severe acute respiratory syndrome coronavirus 2 (SARS‐CoV‐2) has been identified through several conventional molecular methodologies that are time‐consuming and require high‐skill labor, apparatus, and biochemical reagents but have a low detection accuracy. These bottlenecks hamper conventional methods from resolving the COVID‐19 emergency. However, interdisciplinary advances in nanomaterials and biotechnology, such as nanomaterials‐based biosensors, have opened new avenues for rapid and ultrasensitive detection of pathogens in the field of healthcare. Many updated nanomaterials‐based biosensors, namely electrochemical, field‐effect transistor, plasmonic, and colorimetric biosensors, employ nucleic acid and antigen–antibody interactions for SARS‐CoV‐2 detection in a highly efficient, reliable, sensitive, and rapid manner. This systematic review summarizes the mechanisms and characteristics of nanomaterials‐based biosensors for SARS‐CoV‐2 detection. Moreover, continuing challenges and emerging trends in biosensor development are also discussed.

## Graphical abstract

Interdisciplinary advances in nanomaterials and biotechnology, such as nanomaterials‐based biosensors, have opened new avenues for rapid and ultrasensitive detection of pathogens in the field of healthcare. Many updated nanomaterials‐based biosensors, namely electrochemical, field‐effect transistor, plasmonic, and colorimetric biosensors, employ nucleic acid and antigen–antibody interactions for severe acute respiratory syndrome coronavirus 2 (SARS‐CoV‐2) detection in a highly efficient, reliable, sensitive, and rapid manner. This systematic review summarizes the mechanisms and characteristics of nanomaterials‐based biosensors for SARS‐CoV‐2 detection. Moreover, continuing challenges and emerging trends in biosensor development are also discussed.

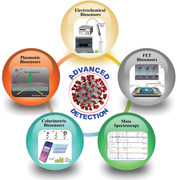

## 1 INTRODUCTION

Severe acute respiratory syndrome coronavirus 2 (SARS‐CoV‐2), the root cause of the current Coronavirus disease 2019 (COVID‐19) pandemic named so on February 11, 2020, by the report of the World Health Organization, has caused more than 6 million deaths worldwide by December 2022. Additionally, SARS‐CoV‐2 variants, from Alpha to Omicron as of late November 2021, are produced by a change and/or mutation in the genome of RNA viruses over time. The majority of people infected with COVID‐19 undergo fever with or without dry cough, chest tightness, dyspnea, and viral pneumonia.^[^ ^1^ ^]^ According to the report from the Centers for Disease Control and Prevention, the symptoms become complicated and potentially fatal in the presence of Alpha and/or Delta variants. Remarkably, the Delta variant (or B.1.617.2) is considered the most contagious variant all over the world. Therefore, testing is a top priority in defending against virus transmission and reducing the number of unreported infections. Moreover, diagnostics are critical in deciding the timely treatment and isolation of detected cases, thereby delaying or preventing the pervasive pandemic. Rapid and reliable diagnostic testing with point‐of‐care (PoC) technologies is advantageous at the initial stage, as experienced in previous viral epidemics, such as the SARS outbreak in 2003, H1N1 in 2009, Middle East respiratory syndrome coronavirus (MERS‐CoV) in 2012, and Ebola in 2015.^[^ ^2^, ^3^, ^4^, ^5^, ^6^ ^]^

Conventional strategies applied for COVID‐19 identification are viral RNA‐based molecular tests. Reverse transcription‐polymerase chain reaction (RT‐PCR), in which viral RNA is reversely transcribed into complementary DNA (cDNA) prior to the cDNA amplification stage, is the gold standard molecular testing strategy for diagnosing COVID‐19.^[^ ^7^, ^8^, ^9^, ^10^, ^11^ ^]^ Signal registration can be accomplished by observing the real‐time reaction, and/or post‐reaction analysis. Additionally, RT‐PCR can be combined with another assay, namely, loop‐mediated isothermal amplification (LAMP),^[^ ^12^, ^13^ ^]^ recombinase polymerase amplification (RPA),^[^ ^14^, ^15^ ^]^ clustered regularly interspaced short palindromic repeats (CRISPR),^[^ ^16^, ^17^, ^18^, ^19^, ^20^, ^21^ ^]^ enzyme‐linked immunosorbent assay (ELISA),^[^ ^22^, ^23^, ^24^ ^]^ and lateral flow immunoassay (LFIA).^[^ ^25^, ^26^ ^]^ However, their applications are a big challenge during the COVID‐19 pandemic, as described in Table 1. All of the aforementioned methods, which have the same deficiency in the timely screening of initial‐stage patients and asymptomatic carriers, are time‐consuming and require complex procedures, necessitating sophisticated equipment and highly skilled workers.

**TABLE 1:** Pros and cons of conventional molecular methodologies for COVID‐19 detection

Assay	PoC	Pros	Cons	Ref.
RT‐PCR	Specialized laboratories and hospitals	The gold standard molecular testing technique for COVID‐19 diagnosis Sensitive and reliable	Well‐trained labor, advanced apparatus, and facilities, expensive reagents, time‐consuming process (∼1 h) False‐positive and false‐negative results	[7, 8, 9, 10, 11]
LAMP	Specialized laboratories and hospitals	Shorter assay time (40 min) compared to RT‐PCR Cost‐effective compared to RT‐PCR due to the elimination of a few steps essential for quantitative RT‐PCR	The complexity of designing the required primers Well‐trained labor	[12, 13]
RPA	Specialized laboratories	Short assay time and high sensitivity	Designing the required primers Well‐trained labor	[14, 15]
CRISPR	Specialized laboratories	A potent and robust molecular diagnostic tool due to the ability of Cas12a and/or Cas13a Specificity can be enhanced by combining primer‐specific amplification	The limitations of amplification techniques and utilized Cas‐enzyme activity may have an impact on the test results Well‐trained and highly skilled labor and sophisticated equipment	[16, 17, 18, 19, 20, 21]
ELISA	Clinics and laboratories	Cost‐effective and short assay time	Test results accurate only after 10–14 days of symptom onset Assay results rely on colorimetric analysis, which has limited sensitivity and quantitative capability	[22, 23, 24]
LFIA	Public	Simplicity, flexibility, speed, cost‐effectiveness, and rapid diagnosis Commercialized test kits Tests require little to no training	Assay results rely on colorimetric analysis Positive result should be confirmed by RT‐PCR	[25, 26]

Nanotechnology is considered an interdisciplinary field with diverse applications. Nanobiotechnology is a new era of nanotechnology that is concerned with the biological activities of nanomaterials, applying biological principles and physicochemical processes to the generation of nanoparticles (NPs) for specific applications, especially nanomaterial‐based biosensors that are used to detect COVID‐19.^[^ ^27^, ^28^ ^]^ NPs have played a vital role in this technology due to their incredibly tiny size and high surface area‐to‐volume ratio that can be controlled by synthesis methods, doping, and hybridization approaches,^[^ ^29^, ^30^ ^]^ which enhance their physicochemical, electrical, mechanical, and optical characteristics. Recently, metal oxides have been of great interest in antimicrobial and cytotoxicity studies.^[^ ^29^, ^31^, ^32^ ^]^ Moreover, diverse nanomaterials, including noble metals (Ag and Au),^[^ ^33^ ^]^ carbon compounds [graphene, graphene oxide (GO), and carbon nanotube (CNT)],^[^ ^34^ ^]^ semiconductors,^[^ ^35^, ^36^ ^]^ and conducting polymers,^[^ ^37^ ^]^ have been exploited in healthcare applications. Sensitivity, specificity, response accuracy, reproducibility toward target elements, economic efficiency, and nontoxicity are all requirements for biosensors.^[^ ^38^, ^39^, ^40^ ^]^ Antigens or antibodies attached to nanomaterials efficiently maintain their bioactivity and counterpart interaction and can be quantified by nanomaterial detection. Therefore, multiplexed nanomaterial‐based sensors for detecting COVID‐19 have been extensively researched. The suggested gadget can successfully differentiate infected cases from control groups as well as COVID‐19 from other lung disorders according to preliminary findings.^[^ ^41^, ^42^, ^43^ ^]^ Nonetheless, the epidemic is still wreaking havoc on public health systems worldwide. Preventing and stopping ubiquitous COVID‐19 transmission by presymptomatic and asymptomatic cases, mainly in the community and at home, requires ultrasensitive and early infection detection.

In this review, we extensively discuss SARS‐CoV‐2 detection based on nucleic acid and antigen–antibody interactions. Nanomaterials functionalized with nucleic acid [DNA, RNA, and synthetic oligonucleotide (aptamer)], antibodies, or antigens have been used for fast, sensitive, specific, and direct detection with exceptional potential. Moreover, biosensors are appropriate for pathogenic virus detection with excellent response/specificity and enable quick tests with reliable, simple procedures and miniaturization. They offer a lot of promise for PoC testing and analysis. Our review elaborates on the interactions, categories, and mechanisms between nanomaterials and viral host cells and discusses advanced developments in the design of diverse biosensors [e.g., electrochemical (EC), field‐effect transistor (FET), plasmonic, and colorimetric biosensors], as well as the bottlenecks and prospects for SARS‐CoV‐2 identification. In addition, we update knowledge regarding the state‐of‐the‐art mass spectroscopy technologies with ultrasensitivity/specificity containing a huge database that can analyze the genome of SARS‐CoV‐2 with existing variants, allowing for evaluating the quality of vaccines to tackle COVID‐19 and similar pandemics in the future.

## 2 PRINCIPLE OF BIOSENSOR DETECTION

Due to the rise of the COVID‐19 pandemic, biosensors have been employed and optimized for rapid test and diagnosis of COVID‐19 patients with significant merits, including high efficiency, flexibility, and specificity.^[^ ^44^, ^45^, ^46^, ^47^ ^]^ Biosensors comprise a biological element to capture the virus and a transducer to convert the captured virus in the sample into a detectable signal; electronic systems subsequently amplify and express the signal for quantitative detection.^[^ ^48^, ^49^ ^]^ Notably, the biorecognition elements could be enzymatic reactions, whole cells, genetic material of virus such as DNA and RNA, and products of patient's immune response system such as antibodies.^[^ ^50^, ^51^ ^]^ Based on the biorecognition element, there are two major mechanisms of biosensors for SARS‐CoV‐2 detection: (i) the detection of nucleic acid using a single complementary strand of DNA/RNA or aptamers and (ii) the detection of antigen/antibodies in patient's samples. The general principle of biosensors used in SARS‐CoV‐2 detection is discussed below.

### 2.1 Nucleic acid‐based detection

**FIGURE 1:**
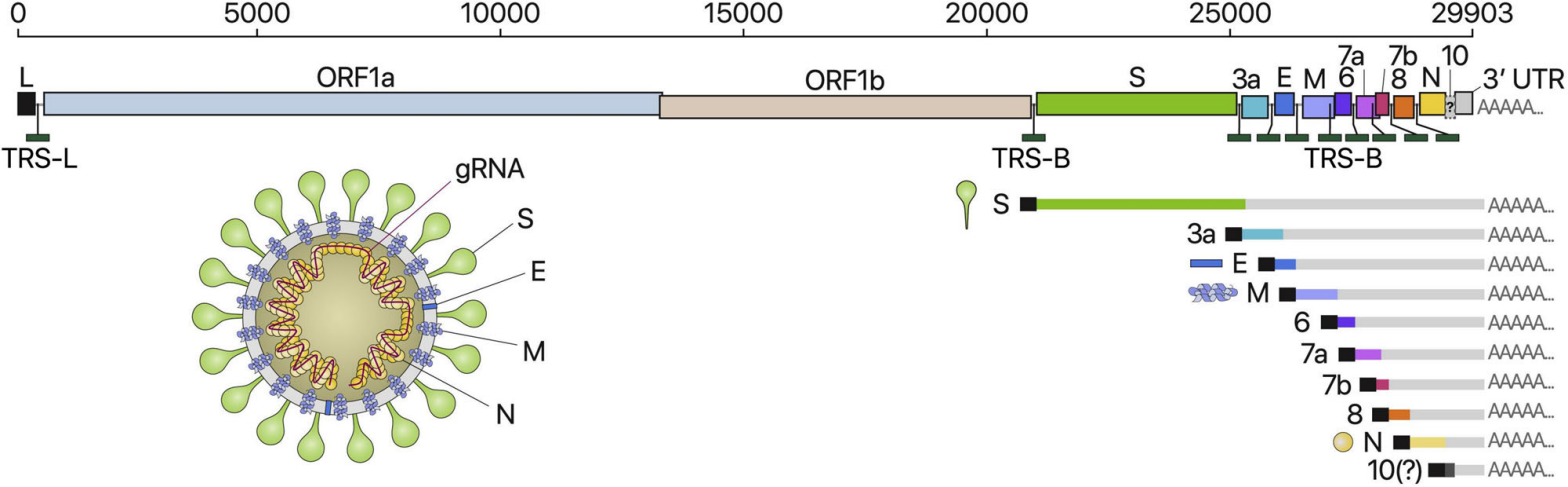
Schematic presentation of the SARS‐CoV‐2 genome organization, the canonical subgenomic mRNAs, and the virion structure. Adapted with permission.^[^ ^52^ ^]^ Copyright 2020, Elsevier

SARS‐CoV‐2 was first isolated and classified as a *Coronaviridae* with its genome as a positive‐sense, single‐stranded RNA of ∼30 kb. According to annotation and analysis, the genome of COVID‐19 comprises several functional genes such as spike glycoprotein (S gene), envelope protein (E gene), matrix protein (M gene), nucleocapsid protein (N gene), and noncoding regions at the end of 5′ and 3′ terminal regions of the gene, as shown in Figure 1.^[^ ^52^ ^]^ Based on this background of viral genetic material, COVID‐19 can be detected early by RT‐PCR using the patient's nasal and throat swab samples.^[^ ^53^ ^]^ In this technique, a pair of primers is utilized to bind specifically to the cDNA of the virus, and a fluorescent readout system is used to detect the viral infection.^[^ ^7^ ^]^ Nevertheless, several false‐positive and false‐negative diagnoses have been recorded during the COVID‐19 pandemic, especially due to overwhelming case loads.^[^ ^11^ ^]^ The development of nanomaterials combined with complementary single‐strand DNA (ssDNA)/RNA or aptasensors tagged with reporters (fluorescent or luminescent) has been researched explosively and applied worldwide to help medical professionals in the screening of overloaded cases effectively during the pandemic.^[^ ^54^, ^55^, ^56^ ^]^ Several cutting‐edge studies on DNA/RNA‐based or aptamer‐based sensors will be summarized in this section.

**FIGURE 2:**
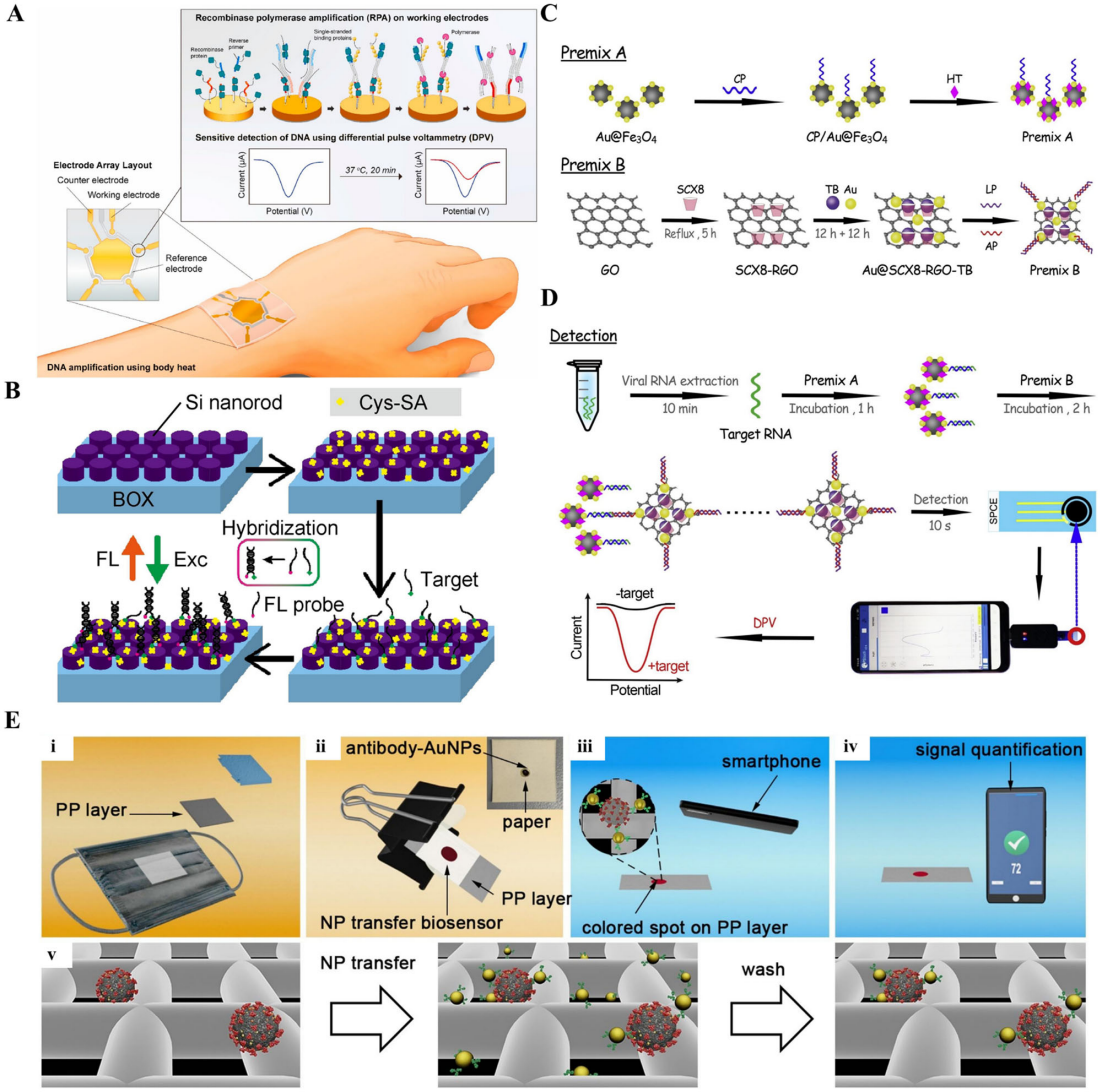
(A) An EC biosensor combined with isothermal amplification. Schematic of the EC biosensor combined with RPA. The RPA reaction occurs on the working electrodes, and amplicon detection is quantified by differential pulse voltammetry. Schematic representation of SARS‐CoV‐2 detection using the EC biosensor. Adapted with permission.^[^ ^54^ ^]^ Copyright 2021, Elsevier. (B) Schematic of DNA detection on the metasurface. Cys‐SA denotes Cys‐streptavidin. Reproduced under the terms of the Creative Common CC BY license.^[^ ^64^ ^]^ Copyright 2021, The Author. (C) Preparation of premix A and B. (D) Process of EC identification using a smartphone. Adapted with permission.^[^ ^55^ ^]^ Copyright 2021, Elsevier. (E) Schematic representation of non‐invasive detection of SARS‐CoV‐2 antigens trapped in patients’ masks Adapted with permission.^[^ ^109^ ^]^ Copyright 2021, Elsevier

EC biosensors have a quick reaction time and can be built at minimal costs because the required instrumentation is simple. They are fabricated by immobilizing nucleic acids (DNA, RNA, or aptamers) on the solid support as an electrode via adsorption, electrostatic interaction, or covalent bonding and are integrated with a physicochemical transducer^[^ ^57^, ^58^ ^]^ to measure changes in electrical characteristics (such as conductance, current, impedance, and potential) induced by the hybridization reaction. EC approaches for nucleic acid analysis have become more feasible with the advent of solid electrodes.^[^ ^59^, ^60^, ^61^ ^]^ Recently, Kim et al. developed a quick and sensitive method for COVID‐19 diagnosis using an EC biosensor integrated with RPA. Using differential pulse voltammetry (DPV), an EC biosensor based on microelectrode array microchips, which includes a reference electrode (RE), counter electrode (CE), and five separate working electrodes (WEs), could detect numerous target genes, as shown in Figure 2A.^[^ ^54^ ^]^ To ensure stability and reproducibility, multi‐array WEs were manufactured by the sequent deposition of Ag and Au NPs under the same conditions. The Au electrode exhibits outstanding binding characteristics with thiol groups via strong Au‐S interaction. Moreover, Au NPs have been extensively utilized to enhance biosensing efficacy owing to many superb characteristics, including high conductivity, chemical stability, and large surface area.^[^ ^62^ ^]^ Therefore, specific primers of RNA‐dependent RNA polymerase (RdRp) and N genes modified with thiol groups can enhance absorption on the WEs. The enhanced adsorption of the amplicons results in a significant drop in current density when the negatively charged amplicons electrostatically interact with the WEs. As a result, the proposed biosensor can capture target genes roughly within 20 min at body temperature, which is shorter than RT‐PCR as there is no requirement for an expensive thermal‐cycler. Moreover, the limit of detection (LoD) for the N gene (3.925 fg/μl) and RdRp (0.972 fg/μl) gene is relatively lower than that of the pristine RPA technique.^[^ ^54^ ^]^

Biomarker fluorescence (FL) detection is a constructed method in biosensor technology and is considered one of the most efficient biosensing approaches with potential for future medical diagnosis.^[^ ^63^ ^]^ However, most of the FL‐improved platforms described to date show poor reproducibility due to the hot spot strategy for achieving large enhanced FL intensity. Hitherto, a specific form of all‐dielectric metasurface can significantly improve FL to detect antigens and antibodies in high reproducibility. The fully dielectric metasurface biosensor contains a silicon‐on‐insulator (SOI) nanorod array and has a significant electromagnetic resonance that amplifies FL emission. In the proof‐of‐concept experiment of the metasurface biosensor, FL detection of ssDNA on the partial sequence of SARS‐CoV‐2 RNA was performed successfully in high throughput detection. First, the tagging molecule is immobilized on the metasurface of SOI nanorods. In this case, Cys‐streptavidin is utilized as a tagging molecule (yellow) because it possibly attaches to SOI nanorods via His‐tags. Second, the target has a biotin label (green) at a specific concentration, flows through a metal surface with polydimethylsiloxane microfluidic chip attached, and is immobilized on the tagging molecule. Finally, ssDNA, a target‐complementary FL‐labeled probe, hybridizes to the target on the metasurface and is captured via an amplified FL signal, as depicted in Figure 2B.^[^ ^64^ ^]^ The resulting biosensor exhibits an LoD of 0.11 fmol/ml without amplification technology.

A super sandwich‐type EC biosensor has sparked considerable interest in the research area of nucleic acid biosensors owing to its high specificity and accuracy.^[^ ^65^, ^66^ ^]^ In principle, the capture probe (CP), targeted sequence, label probe (LP), and auxiliary probe (AP) form this sort of biosensor. The 5′ and 3′ binding sites of the target sequences are correspondingly paired to CP and LP, whereas the 5′ and 3′ domains of the AP contain matching sequences with two distinct LP areas. Consequently, the CP and the LP may be used to identify sequences, and the AP hybridizes with the LP several times to generate large concatemers, leading to great selectivity. As shown in Figure 2C,^[^ ^55^ ^]^ the following processes were used to design and build the super sandwich‐type biosensor for COVID‐19 diagnosis: (i) Thiol‐labeled CPs were fixed on Au@Fe_3_O_4_ surface to generate CP/Au@Fe_3_O_4_ nanocomposites; (ii) *p*‐sulfocalix[8]arene‐toluidine blue (SCX8‐TB) host‐guest recognition, stable and capable of boosting the amplification ability of guest molecules owing to a highly rigid and well‐defined cavity, was fixed on reduced graphene oxide (RGO) to construct Au@SCX8‐TB‐RGO‐LP bioconjugate; (iii) the sandwich configuration of “CPtarget‐LP” was subsequently developed; and (iv) the AP was added to create lengthy concatemers. Using an EC biosensor with a smartphone as shown in Figure 2D,^[^ ^55^ ^]^ researchers created a plug‐and‐play technique for detecting SARS‐CoV‐2 in samples from different clinical specimens in a sensitive, accurate, and quick manner without RNA amplification and reverse transcription. The resulting calibration curve revealed linearity in a range of 10^−17^–10^−12^ M with an LoD of 3 aM that exhibited a relationship between current and target amount. The suggested biosensor demonstrated excellent response due to the high conductivity of Au NPs and reduced GO, and the ability to concentrate the signaling TB species based on SCX8 supramolecular recognition. This approach exhibited viral RNA‐detectable ratios greater than that of RT‐qPCR (85.5% and 46.2% vs. 56.5% and 7.7% from 25 infected cases and 8 recovery cases, respectively). The LoD was 200 copies/ml, which is the lowest value found in any reports of COVID‐19 detection to date.^[^ ^55^ ^]^

### 2.2 Immuno‐based detection

Immunosensors are biosensing devices that use affinity ligands to couple immunoassay responses to compatible transducers. They have advanced rapidly in recent decades, with a wide range of applications and detection methods.^[^ ^67^, ^68^, ^69^, ^70^, ^71^, ^72^, ^73^ ^]^ In the immunosensors' main operating concept, the specified immunochemical reaction of antibodies (or antigens) attached on a transducer to antigens (or antibodies) in the experimental solution provides quantitative signals that dynamically fluctuate with the levels of analytes of interest.^[^ ^74^, ^75^, ^76^ ^]^ Different forms of bonding are involved in the exceedingly specific response between various domains of an antibody and the epitopes of an antigen, namely hydrophobic and electrostatic contacts, and noncovalent bonding.^[^ ^77^, ^78^, ^79^, ^80^, ^81^, ^82^, ^83^, ^84^, ^85^, ^86^ ^]^ The antigen–antibody interaction is bidirectional, and the established complex would separate depending on the surrounding environment due to the relative weakening of the affinity of the complex (e.g., pH and ion strength).^[^ ^87^, ^88^, ^89^, ^90^ ^]^ The antigen–antibody affinity is evaluated by their affinity constant called K,^[^ ^91^, ^92^, ^93^, ^94^, ^95^, ^96^ ^]^ which fluctuates from 5 × 10^4^ to 1 × 10^12^ L/mol. The selectivity and specificity of an immune‐based sensor can be demonstrated by the high value of affinity.

For COVID‐19 detection, in particular, the design of immunosensors generally includes three main parts. First, antigens and/or antibodies act as biological recognition elements to detect antibodies in patient samples/or viral antigens. Second, the antigens/antibodies are attached to the physicochemical transducer surface, including an EC, optical, or thermal transducer. Here, the interactions of immune response elements stimulate physiochemical transducers expressing measurable signals. Finally, the output from transducers is amplified and computerized by the electronic part via several devices, including impedance, amperometric, fluorescence, luminescence.^[^ ^97^, ^98^, ^99^, ^100^, ^101^, ^102^, ^103^, ^104^, ^105^ ^]^ The combination of microfluidics and nanotechnology has provided a high‐performance and high‐throughput sensing for the next generation of immunosensor, especially for rapid SARS‐CoV‐2 detection. SARS‐CoV‐2 utilizes angiotensin‐converting enzyme II (ACE2) as a cellular entrance receptor via the S1 subunit of S gene with high affinity, according to recent research; ACE2 is recognized as SARS‐CoV host cell receptor to animal cells.^[^ ^106^, ^107^, ^108^ ^]^ Based on this evidence, many studies involving immunosensors for SARS‐CoV‐2 detection have been conducted, applying nanomaterial which has accelerated and improved the global pandemic.

Noninvasive SARS‐CoV‐2 detection is a novel method for detecting captured viral antigens trapped in medical masks worn by infected people using Au NP‐based biosensor, as depicted in Figure 2E.^[^ ^109^ ^]^ The proposed biosensors are made of a polymer‐modified filter paper containing antibody‐decorated Au NPs. At dry conditions, the polymer prevents paper–nanoparticle interaction, ensuring total Au NPs are liberated after the injection of liquid. Interestingly, the transfer of Au NPs from the biosensor to the mask emits colorimetric signals in the appearance of COVID‐19 IgG that can be obtained using a smartphone app. Sample collection needs 30 min of wearing the attached mask, while the complete assay time is <10 min. The resulting biosensor exhibited a sensitivity of 96.2% and specificity of 100% in testing mild or asymptomatic patients. This approach is affordable, simple, and readily accessible to implement anywhere with excellent sensitivity and specificity, regardless of any symptoms in infected individuals.

**FIGURE 3:**
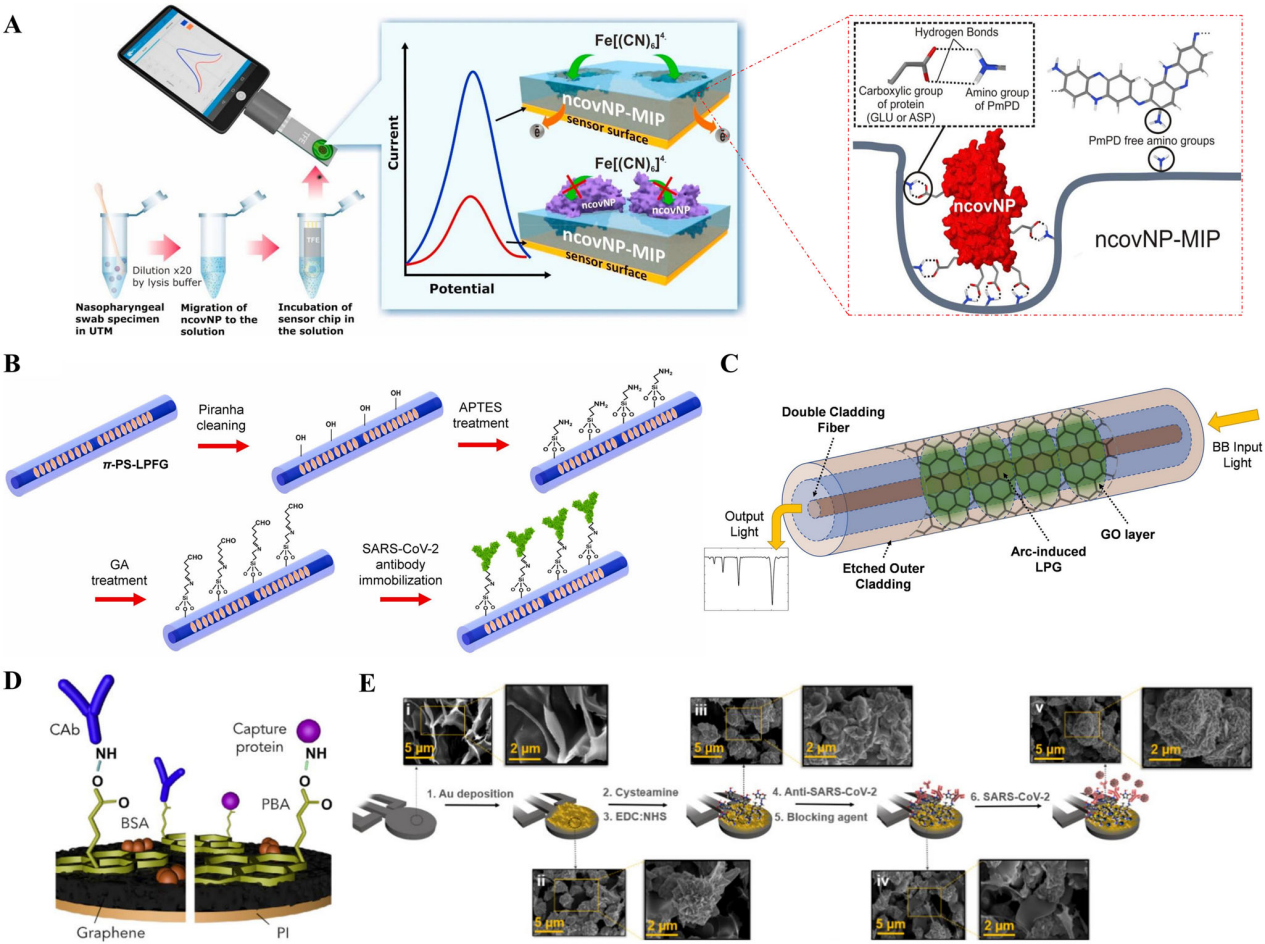
(A) COVID‐19 testing principle by nconNP sensor. Adapted with permission.^[^ ^113^ ^]^ Copyright 2021, Elsevier. (B) Schematic diagram of SSA immobilization steps through surface functionalization of fabricated PS‐LPFG: Piranha cleaning, APTES treatment, GA activation, and SSA immobilization. Adapted with permission.^[^ ^115^ ^]^ Copyright 2021, Elsevier. (C) Schematic view of the optical transducer based on LPG in DCF coated with GO. Adapted with permission.^[^ ^117^ ^]^ Copyright 2021, Elsevier. (D) Scheme detailing the methodology developed for the covalent attachment of the corresponding bioreceptor for the specific capture of the target analytes SARS‐CoV‐2 NP and CRP (left), and IgG and IgM isotypes against SARS‐CoV‐2 S1 protein (right). PBA, 1‐pyrene butyric acid; BSA, bovine serum albumin; CAb, capture antibody; PI, polyimide. Adapted with permission.^[^ ^121^ ^]^ Copyright 2020, Elsevier. (E) Representation of each experimental step with scanning electron microscopy (SEM) images showing the morphology at high magnification (scale bar; 2 μm) and low magnification (scale bar; 5 μm). Adapted with permission.^[^ ^122^ ^]^ Copyright 2021, American Chemical Society

Molecularly imprinted polymers (MIPs) are produced by processes that allow synthetic antibodies, receptors, or specific aptamers to become recognition sites for binding to bacteria, viruses, mammalian cells, or other biomolecules, solving problems related to specific biological systems. This method has been applied as a template for generating specific target sequences by immobilizing synthetic polymers via covalent, noncovalent, electrostatic, or metal center coordination binding interactions. Subsequently, the generation of binding sites after the template removal is complementary to the specific recognition in terms of size, shape, and chemical functionality, allowing these molecules to be selectively recognized.^[^ ^110^, ^111^ ^]^ An amphiphilic copolymer was used as a macromonomer to develop protein sensing. Copolymer poly(DMA*‐co‐*HEA*‐co‐*St) (UPDHS) self‐assembles with protein template under UV irradiation to produce protein imprinted polymeric NPs. To maintain the internal structure of the protein, linear macromolecular chains were employed to construct a 3D shape around the molecule, resulting in recognition cavities. The NPs were then fixed as an MIP sensing platform on the transducer surface. Finally, the resulting MIP coating was exposed to UV light to ensure that the recognition cavities remained stable during UV curing. The sensor has superb specificity in a good linear range of 10^−14^–10^−9^ mg/ml and a lower LoD than other sensors. This technology provides a new and simple way to synthesize receptors for label‐free and protein recognition.^[^ ^112^ ^]^ Abdul et al. pioneered an MIP‐based EC sensor, which was fabricated by modifying Au thin‐film with SARS‐CoV‐2 nucleoprotein (ncovNP)‐MIP film produced from poly‐*m*‐phenylenediamine (PmPD), is a one‐use chip based on Au electrode for identifying the ncovNP. The Au chip integrated with a portable galvanometer is driven by software in an electronic device, specifically capturing ncovNP with a redox pair driven by DPV, as shown in Figure 3A.^[^ ^113^ ^]^ The galvanostat studies a decrease in charge transfer intensity of the [Fe(CN)_6_]^3−^/[Fe(CN)_6_]^4−^ redox probe to the Au chip via the ncovNP‐MIP film. After aging the sensor in a specific solution, the film rebounds the ncovNP target and the charge transfer is effectively blocked, resulting in a current drop that coincides with the virus protein concentration in the nasopharyngeal swab specimens. While Au thin‐film modified by a 4‐ATP monolayer almost did not affect anodic/cathodic current peaks, implying that this short thiol was too thin to efficiently trap electron transfer on the electrode surface, the redox current peaks were substantially reduced after the subsequent binding of ncovNP via 3,3′‐dithiobis [sulfosuccinimidyl propionate]. Despite this, PmPD development may be initiated at a low potential (0.6 V against Ag/AgCl/KCl), comparable to that of a bare Au‐electrode. The sensor responded linearly to ncovNP in the lysis buffer at concentrations ranging 2.22–111 fM, with an LoD of 15 fM and a limit of quantitation (LOQ) of 50 fM. It also enabled ncovNP differentiation from other proteins [S protein, bovine serum albumin (BSA), CD48 protein, and Hepatitis C virus] that interfered with ncovNP.

Long‐period fiber grating (LPFG) is represented by a cyclic modulation of the refractive index of fiber optic cores with periods of hundreds of micrometers. LPFG is superior to other technology platforms owing to several merits, including lightness, miniaturization, polarization independence, real‐time and remote sensing abilities, simple operation, and resistance to electromagnetic interference, and innate response to surrounding medium refractive index (SMRI) varies. The influence of SMRI variation on LPFG resonance wavelength is highlighted, as a change in the wavelength is considered a biosensing indicator that is driven by biological reactions on the fiber surface. The wavelength‐encoded signals are more dependable than an intensity‐based interrogation technique, which undergoes unanticipated power variations produced by exterior disturbances.^[^ ^114^ ^]^ To obtain good specificity to desired analytes, the LPFG surface is modified using a selective bio‐layer that covalently binds biological recognition elements to the surface. The bio‐functionalized LPFG facilitates the generation of a biosensor based on the LPFG that is capable of detecting trace amounts of analytes with great sensitivity to SMRI change driven by biological activity. SARS‐CoV‐2 S protein immobilization on functionalized phase‐shifted (PS)‐LPFG at room temperature is depicted in Figure 3B.^[^ ^115^ ^]^ To eliminate organic impurities, the as‐prepared PS‐LPFG was treated for 30 min with piranha solution comprising concentrated H_2_SO_4_ and H_2_O_2_ in a ratio of 3:1. The PS‐LPFG was properly washed thrice with deionized (DI) water after the cleaning step. (3‐Aminopropyl) triethoxysilane (APTES) was used as a hydrophilic silane coupling reagent to produce functional amine (─NH_2_) groups on the PS‐LPFG surface by dipping PS‐LPFG into 5% (v/v) APTES/ethanol (EtOH) solution for 2 h. Subsequently, the PS‐LPFG was consecutively washed multiple times with EtOH and DI water to fully eliminate the remaining chemicals before 1 h thermal annealing at 120°C. Following the creation of the amine‐functionalized surface, the PS‐LPFG was treated for 3 h with a 2% (v/v) glutaraldehyde solution to produce aldehyde (─CHO) groups that can be labeled and cross‐linked with ─NH_2_ groups of S protein after exposure to 100 μg/ml S protein for 3 h.^[^ ^115^ ^]^ However, the ideal size of Au NP labels (20, 40, and 60 nm) was examined to produce high sensitivity and low LoD for N protein detection. Consequently, citrate‐capped Au NP labels (size 40 nm) produced an LoD of 2.5 ng/ml within 10 min of read‐out time. In addition, preclinical investigations and the development of a PoC read‐out device are in the plans.^[^ ^116^ ^]^ A thin coating of GO was formed around the LPFG portion to generate functional groups for the biological recognition element immobilized by covalent bonds, as shown in Figure 3C.^[^ ^117^ ^]^ The outside cladding of the LPFG has a greater refractive index than the inner cladding, allowing some cladding modes to be steered in the outer ring‐shaped cladding without interfering with the core mode. However, the transition phenomena from outer to inner cladding of such modes can be generated by chemical etching of the LPFG (i.e., lowering the external cladding thickness). The resulting optical biosensor exhibited a wide range of clinically relevant C‐reactive protein (CRP) concentrations (1 ng/ml to 100 μg/ml) that was captured by real‐time analysis, with an LoD of 0.15 ng/ml in serum. The gadget was run by a cutting‐edge microfluidic system for fiber‐optic biosensors, which has exhibited the best result so far for CRP recognition as a biomarker of COVID‐19.

Graphene is a high‐surface‐area, highly conductive, and stable substance.^[^ ^118^ ^]^ However, because it lacks chemically reactive functional groups, further NP functionalization may be required. Due to its potential to boost catalytic activity and inhibit viruses, bacteria, and fungi photothermally or by producing reactive oxygen species (ROS), NPs have become generally employed in healthcare applications.^[^ ^119^, ^120^ ^]^ Figure 3D depicts functionalization and modification of the graphene surface for the covalent attachment of specific receptors.^[^ ^121^ ^]^ 1‐Pyrenebutyric acid (PBA) comprises a pyrene group and a carboxylic acid (─COOH) group to functionalize the graphene surface by strong π‐stacking interactions. The functional units of PBA molecules can create affinity‐based biosensor platforms by covalent bonding of ─COOH sites of PBA with ─NH_2_ sites of specific capture receptors, including capture antibodies (CAb) or proteins. The presence of BSA hampers unexpected adsorption of molecules that is related to assay arrangement by blocking unreactive sites. Functionalization of graphene surface is used to capture COVID‐19 S protein. Three‐electrode design includes an Au nanostructure (AuNS)‐functionalized laser‐scribed graphene (LSG) WE, an LSG‐based CE, and an RE. Original LSG has insufficient functional bindings for essential antibody attachment. Figure 3E demonstrates the preparation procedure for the LSG/AuNS immunosensor.^[^ ^122^ ^]^ First, cysteamine was loaded onto the surface of the WE subsequent to Au deposition, leading to free ammonium groups attachment on the surface. The following step treated the cysteamine‐coated LSG/AuNS with EDC: NHS that activated ─COOH functional sites on the electrode surface allows for robust and quick affinity binding to biorecognition components via amino linkers. Consequently, the S protein with a high concentration of ─COOH groups was attached to the surface, trapping free ammonium groups linked to the surface. Since the S protein detection, BSA was adsorbed on the surface to reduce nonspecific interactions and ensure the stability of sensing efficacy in complex matrices. Finally, a highly porous 3D‐LSG structure sensor integrated with a PoC device is achieved for easily detecting SARS‐CoV‐2 from serum samples. To promote an entirely optimized PoC diagnostic tool, the LSG/AuNS electrode needs to be further improved. Despite this, the proposed sensor system provides a stable alternative platform suitable for future applications.

A rapid, single‐step, simple, and highly accurate diagnostic platform is still a challenge for future epidemic control, particularly in areas with limited medical supplies. Magnetic particle spectroscopy (MPS) was applied to capture specific S and N proteins that were used as biomarkers for COVID‐19. The higher harmonics of magnetic NPs (MNPs) are used as a gauge of the NPs’ binding states in this approach, which tracks their dynamic magnetic responses. These NPs operate as nanoprobes that precisely bind to specific targets (S and N proteins) and create NP clusters by attaching polyclonal antibodies onto MNP surfaces. This binding event results in measurable changes in higher harmonics, which quantitatively and qualitatively identify the specific analytes in the liquid phase. As a result, the LoD of S and N proteins sequentially corresponds to 1.56 nM (∼125 fmoles) and 12.5 nM (∼1 pmole).^[^ ^123^ ^]^ By simply modifying surface functional groups on MNPs, this MPS platform, in combination with the single‐step, NP clustering‐based testing technique, is innately adaptable and enables the identification of diverse disease biomarkers.

## 3 ADVANCED STRATEGIES FOR BIOSENSOR DETECTION

COVID‐19 has been ubiquitous and become an increasingly concerning situation with multiple viral variants that have extremely fast transmission and weaken the effect of the COVID‐19 vaccine, leading to a high risk of death versus recorded viral diseases in history. Therefore, timely and quick diagnosis with excellent sensitivity and specificity is the top goal and a big challenge to prevent future waves of outbreaks from COVID‐19 or another virus. Recently updated strategies are summarized in Table 2.

**TABLE 2:** Recent biosensors used for COVID‐19 detection

Biosensor	Material	Target	LoD	Assay specimen	Assay time	Ref.
EC	Au electrode	RdRp gene	0.972 fg/μl	Body temperature	20 min	[54]
		N gene	3.925 fg/μl			
EC	Modified Au thin film	N protein	15 fM	Nasopharyngeal swab	30 min	[113]
EC	ssDNA‐functionalized Au NPs/graphene	N gene	6.9 copies/μl	Nasal swab or saliva	5 min	[132]
EC	Modified Au electrode	Aptamer	10 pfu/ml	Exhaled breath condensate	10 min	[144]
FET	Carbon nanotube	S1 protein	4.12 fg/ml	Saliva	2–3 min	[159]
FET	AuNP‐decorated graphene	RdRp gene	0.37–3.99 fM	Throat swab and serum	2 min	[153]
FET	S1 protein‐functionalized graphene	Spike antibody	2.6 aM	Clinical serum	< 2 min	[154]
FET	S protein‐binding Au electrode	IgG	1–10 fM	Saliva and serum	5 min	[158]
Plasmonic	Labeled Au NPs	N protein	2.5 ng/ml	Urine	10 min	[116]
Plasmonic	GO‐coated LPFG	CRP	0.15 ng/ml	Serum	30 min	[117]
Plasmonic	Au nanospikes	S protein	∼0.5 pM (0.08 ng/ml)	Blood	30 min	[161]
Plasmonic	Au nanoislands chip	RdRp, ORF1ab, and E genes	0.22 pM	Throat/nasal swabs and sputum	< 15 min	[162]
Plasmonic	Au nanoislands	RNA	0.275 ± 0.051 fM	Nasopharyngeal swabs	30 min	[166]
Colorimetric	Modified Au NPs	IgG	3 ng/ml	Respiratory	< 10 min	[109]
Colorimetric	Functionalized Au NPs	Cytokines (IL‐6 and IL‐10)	10^−3^ pg/ml	Blood and respiratory	10 min	[168]
Colorimetric	Au NPs‐cysteamine‐ACE2	S protein	0.154 pg/ml	Nasopharyngeal/oropharyngeal	5 min	[174]

### 3.1 Electrochemical biosensors

Electrochemical (EC) biosensors refer to biosensor devices that combine the EC sensor with the specific recognition of biomolecules. Quantitative RT‐PCR has been widely used worldwide for COVID‐19 diagnosis through recognition of the SARS‐CoV‐2 viral nucleic acid. Because of the high accuracy and specificity, it is considered the gold standard for COVID‐19 diagnosis.^[^ ^124^, ^125^, ^126^, ^127^, ^128^ ^]^ Yet, its long processing time and requirements for professional operators hinder the potential as a PoC diagnostic technology.^[^ ^129^, ^130^ ^]^ In comparison, EC biosensors have the merits of miniaturization and portability. Besides, they are highly sensitive and specific and can be produced with simple structures at a low cost.^[^ ^131^ ^]^ This allows for a decentralized diagnostic test or even long‐term monitoring of biomarker levels for the early warning of COVID‐19.^[^ ^54^, ^55^, ^132^, ^133^, ^134^, ^135^, ^136^, ^137^, ^138^ ^]^

**FIGURE 4:**
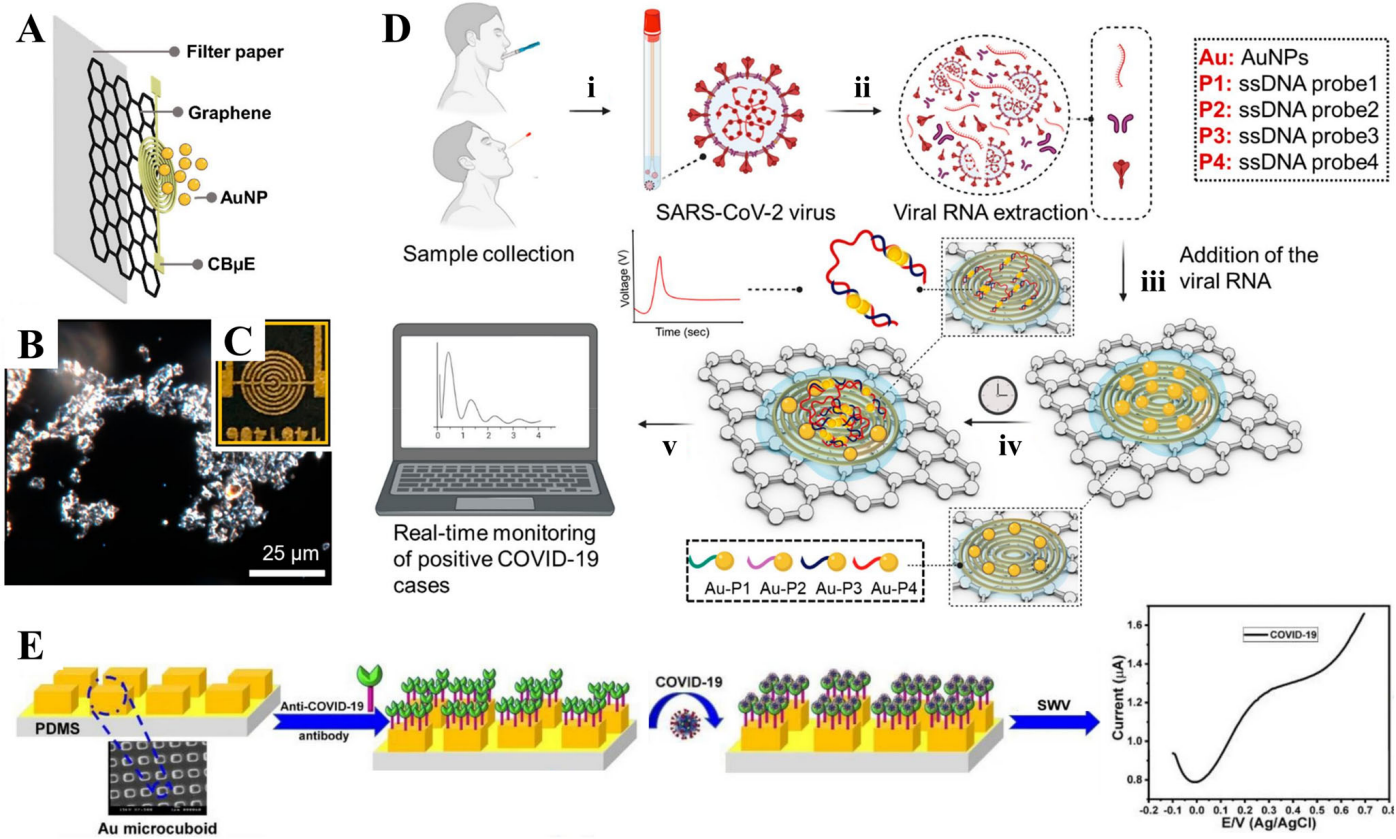
(A–D) A graphene‐based EC sensor for detecting SARS‐CoV‐2 genetic material. Reproduced with permission.^[^ ^132^ ^]^ Copyright 2020, American Chemical Society. (E) A gold‐patterned EC biosensor for recognizing SARS‐CoV‐2 spike protein. Reproduced with permission.^[^ ^134^ ^]^ Copyright 2021, Springer

Alafeef et al. have reported a paper‐based EC sensor chip for detecting nucleocapsid phosphoprotein (N‐gene) of SARS‐CoV‐2. A schematic diagram of the EC sensor structure is illustrated in Figure 4A.^[^ ^132^ ^]^ The biosensor was achieved by the following three steps: (i) Graphene was coated on the filter paper to obtain a conductive film; (ii) highly specific antisense oligonucleotide (ssDNA) probes were modified on the surface of Au NPs; and (iii) Au NPs were deposited on the graphene film with a predefined design. The dark‐field image of Au NPs on the graphene film and the optical image of the biosensor are depicted in Figures 4B and 4C, respectively. The operation of the biosensor involves five steps (Figure 4D):^[^ ^132^ ^]^ (i) collecting nasal swab or saliva samples from patients; (ii) extracting SARS‐CoV‐2 RNA; (iii) adding extracted RNA onto the graphene‐ssDNA‐Au NP platform; (iv) incubation for 5 min; and (v) recording changes in the digital EC signal. Through validation with SARS‐CoV‐2 infected Vero cells and clinical samples, the biosensor demonstrated significant advantages of accurate and rapid diagnosis (5 min incubation time), high sensitivity [231 (copies/μl)^−1^] with a low LoD (6.9 copies/μl).

EI‐Said et al. designed a sensitive EC biosensor with patterned Au microstructures for recognition of the SARS‐CoV‐2 S protein. The schematic diagram of the preparation strategy and detection principle is shown in Figure 4E.^[^ ^134^ ^]^ The sensing function was achieved by immobilization of the IgG1 antibodies on the Au micro cuboid as probes for monitoring the SARS‐CoV‐2 S protein. The output signals were acquired using cyclic voltammetry (CV) and square wave voltammetry (SWV) techniques. The oxidation peaks indicate the bonding between IgG1 antibody and SARS‐CoV‐2 S protein. The biosensor can detect SARS‐CoV‐2 S protein in phosphate‐buffered saline (PBS) solution over a wide concentration range (5–100 pmol/L) and a low LoD of 276 fmol/L. Moreover, the biosensor was also confirmed to show a sensitive response to the real nasal swab sample, enabling the fast and direct diagnosis of COVID‐19 without complicated sample preparation steps.

**FIGURE 5:**
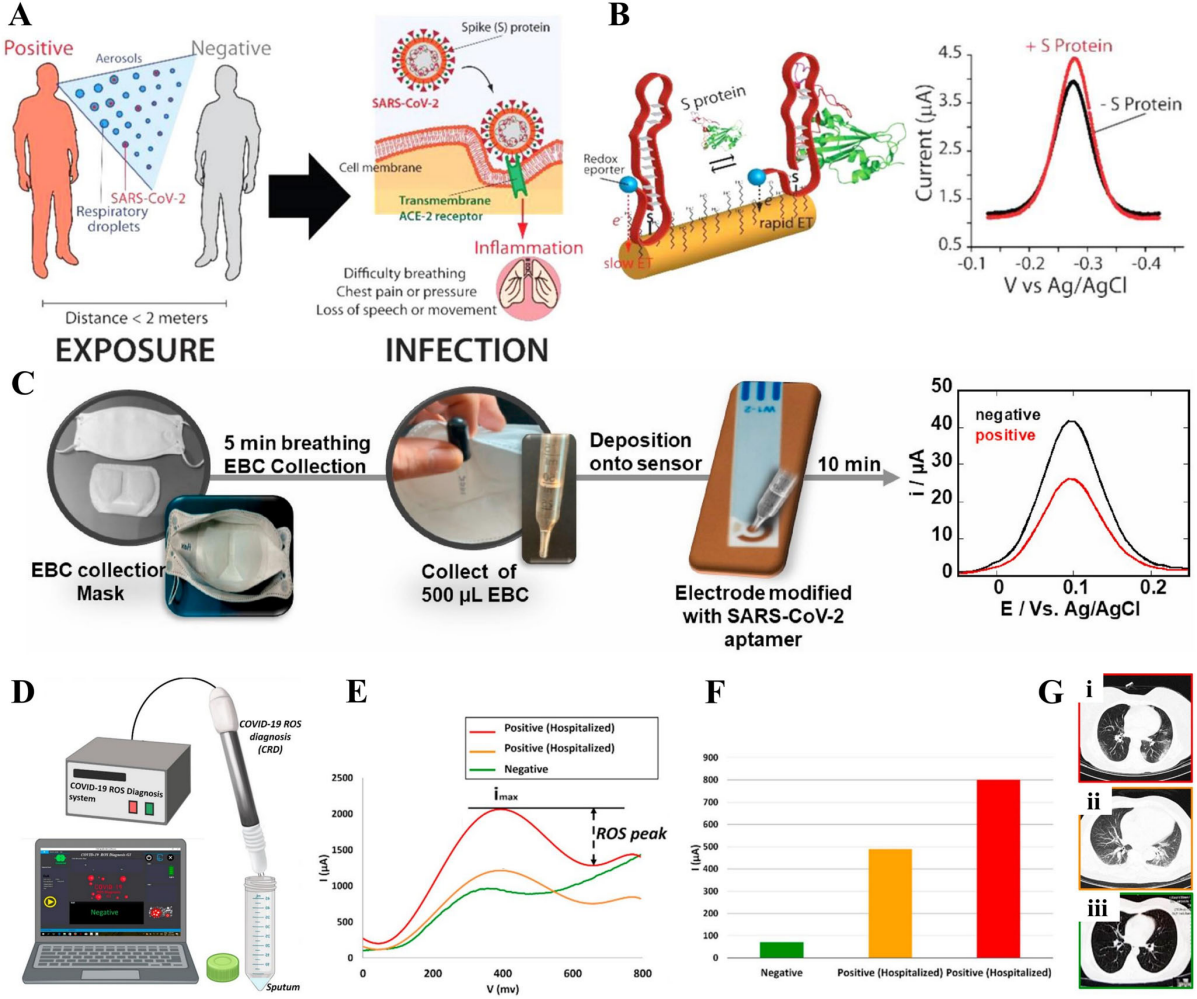
(A,B) An EC aptamer‐based sensor toward fast detecting SARS‐CoV‐2 spike protein. Reproduced with permission.^[^ ^135^ ^]^ Copyright 2021, American Chemical Society. (C) An aptamer‐based EC biosensor using EBC collected by face mask for recognition of SARS‐CoV‐2 S protein. Reproduced with permission.^[^ ^144^ ^]^ Copyright 2021, Elsevier. (D–G) An EC sensor for detecting ROS levels in a sputum sample. Reproduced with permission.^[^ ^136^ ^]^ Copyright 2020, Elsevier

Idili et al. developed an EC aptamer‐based (EAB) sensor for the selective identification of SARS‐CoV‐2 S protein. As described in Figure 5A,^[^ ^135^ ^]^ the rise of the SARS‐CoV‐2 virus relies on the transmission of virus‐containing droplets and aerosols from person to person.^[^ ^129^, ^139^ ^]^ After being inhaled by the infected person, the SARS‐CoV‐2 virus travels deeply into the lungs. The ACE2 receptor situated on the cell surface is the gateway for the virus to enter the host cell.^[^ ^139^ ^]^ The virus binds to cells through its membrane spike (S) proteins that are recognized by the ACE2 receptor. From here, the virus replication cycle starts. In this work, changing target concentration will alter the conformation of the aptamer on the gold electrode. This leads to the relative position change of the redox reporter molecule to the electrode surface and generates a measurable EC signal (Figure 5B).^[^ ^135^ ^]^ The EAB sensor shows clinical potential as a rapid (as fast as 15 s) and simple‐step method for SARS‐CoV‐2 identification through recognition of targets in untreated samples, such as serum and artificial saliva. The high specificity of the EAB sensor is also verified by demonstrating its ability to differentiate between other viral targets.

The kind and technique of sampling are key bottlenecks during patient screening. Collecting nasopharyngeal swabs, saliva, or blood in sterile containers is the current sampling method.^[^ ^140^, ^141^ ^]^ These sampling procedures (nasal swabs and needles) can be uncomfortable for patients and, in certain circumstances, need additional processing steps to isolate the few viruses found in saliva. However, aerosol or filter samplers depend on the long‐term enrichment process after sample collection, resulting in viral collection and enrichment that is time‐dependent.^[^ ^142^ ^]^ To prevent COVID‐19 transmission, governments have mandated the wearing of protective masks in public and at work. Protective masks are considered wearable collectors that are easy‐to‐use and cost‐effective for COVID‐19 diagnosis. This noninvasive platform is built into protective masks to capture airborne viruses in exhaled breath throughout the duration of use.^[^ ^143^ ^]^ To simulate airborne dispersion, the pathogen was enriched by spraying a viral sample into the collector; the enriched pathogen was subsequently extracted for additional analytical assessment with necessary chemicals. However, this method requires 4 h to obtain an accurate COVID‐19 diagnostic result, limiting the practical application. Exhaled breath condensate (EBC) can be considered a significant alternate specimen type for PoC screening of COVID‐19, given the increased requirement for patient‐collected samples. A novel concept based on EBC is easily obtained using a mask‐based sampling device. An EAB biosensor with a modular architecture can be subsequently used, allowing rapid COVID‐19 diagnosis.^[^ ^144^ ^]^ As displayed in Figure 5C,^[^ ^144^ ^]^ a specially designed face mask was used for the collection of EBC. The face mask with a Teflon surface was cooled in a freezer in advance before being worn for 5 min for EBC collection. Subsequently, the EBC was collected using a pipette and deposited onto the SARS‐CoV‐2 specific aptamer modified Au electrode of the EC biosensor, which is capable of detecting various SARS‐CoV‐2 variants at concentrations as low as 10 pfu ml^−1^ in cultured virus suspensions. EBC collection for 5 min and incubation for 10 min endowed the biosensor with a fast and PoC approach for COVID‐19 screening.

Miripour et al. introduced an EC sensor for early COVID‐19 diagnosis by monitoring the ROS levels in sputum.^[^ ^136^ ^]^ As shown in Figure 5D,^[^ ^136^ ^]^ the sensing part of the COVID‐19 ROS diagnosis system comprised functionalized multi‐wall CNT‐coated three needle electrodes. After immersing the probe head in the sputum solution and initiating the test, the ROS level by CV was outputted in 30 s. The ROS CV peaks can be explained by the interaction between ROS released by viral‐infected lung epithelium and the CNT electrode. CV test results of two COVID‐19 positive patients showed much higher ROS peaks (>490 μA) compared with the negative case (70 μA), as depicted in Figure 5E,F. Moreover, the correlation between ROS peaks and CT image results has been verified through the direct detection of hazy patches in both lobes of the lung of confirmed COVID‐19 patients (Figure 5Gi,ii).^[^ ^136^ ^]^ The EC sensor shows high accuracy and sensitivity of 97% in 30 s in comparison with clinical diagnosis results of more than 140 cases. The sensor shows a promising aspect in the rapid screening of patients before CT tests for reducing the burden of testing for COVID‐19 diagnosis.

Electrochemiluminescence (ECL) is a novel labeling technology that is superior to radio‐, fluorescence‐, enzyme‐, and chemiluminescence‐immunoassays. It is a sensitive, fast, and steady, solid‐state labeling immunoassay.^[^ ^145^, ^146^, ^147^, ^148^ ^]^ ECL, a unique chemiluminescent reaction led by EC properties on the surface of an electrode, is a complete integration of two strategies, including chemiluminescence and electrochemistry.^[^ ^149^, ^150^ ^]^ Conventionally, an Au electrode of the ECL biosensor is constructed using linear ssDNA or double‐stranded DNA (dsDNA) to detect SARS‐CoV‐2 nucleic acids.^[^ ^151^, ^152^ ^]^ However, to improve the stability and sensitivity of the biosensor, Fan et al. modified the ECL biosensor on the Au electrode using a DNA tetrahedron (DT) probe as a skeleton that replaces ssDNA and/or dsDNA, which is one of the programmable scaffolds, and enhances the robustness of biosensors to recognize RdRp‐COVID genes.^[^ ^56^ ^]^ The reaction induced by entropy that was designed on the tetrahedral fixed electrode surface after a three‐stranded complex (S3) was generated subsequent to hybridization of sequence S1 and S2 on the DNA‐captured DT probe. As a result, a very high ECL signal was recognized by great amounts of final products on the electrode, demonstrating excellent target recognition. DT‐based ECL sensor has been proven to substantially enhance the specificity and sensitivity for SARS‐CoV‐2 detection with an LoD of 2.67 fM. Moreover, this approach has executed RdRp‐COVID identification in serum specimens, which provides a dependable and achievable sensing platform.

### 3.2 Field‐effect transistor biosensors

A field‐effect transistor (FET) is a three‐terminal device composed of source, gate, and drain electrodes. The operating principle of an FET can be explained as follows. The gate‐source voltage change induces variation of the carrier concentration in the semiconductor channel; thus, the current between the source and drain electrode can be controlled. The FET has merits such as ultra‐high input impedance (10^7^∼10^15^ Ω) and low energy consumption. FET‐based biosensors refer to biosensors produced by combining biotechnology and transistor technology.^[^ ^153^, ^154^, ^155^, ^156^, ^157^, ^158^ ^]^ When target molecules bind to the FET gate the carrier concentration of the semiconductor material changes, inducing a change in the conductance of the FET channel. By monitoring the current change between source and drain electrode, the analyte's presence or even its concentration can be deduced. FET biosensors have inherent properties of high sensitivity and can be used for the detection of trace amounts of analytes.^[^ ^156^ ^]^

**FIGURE 6:**
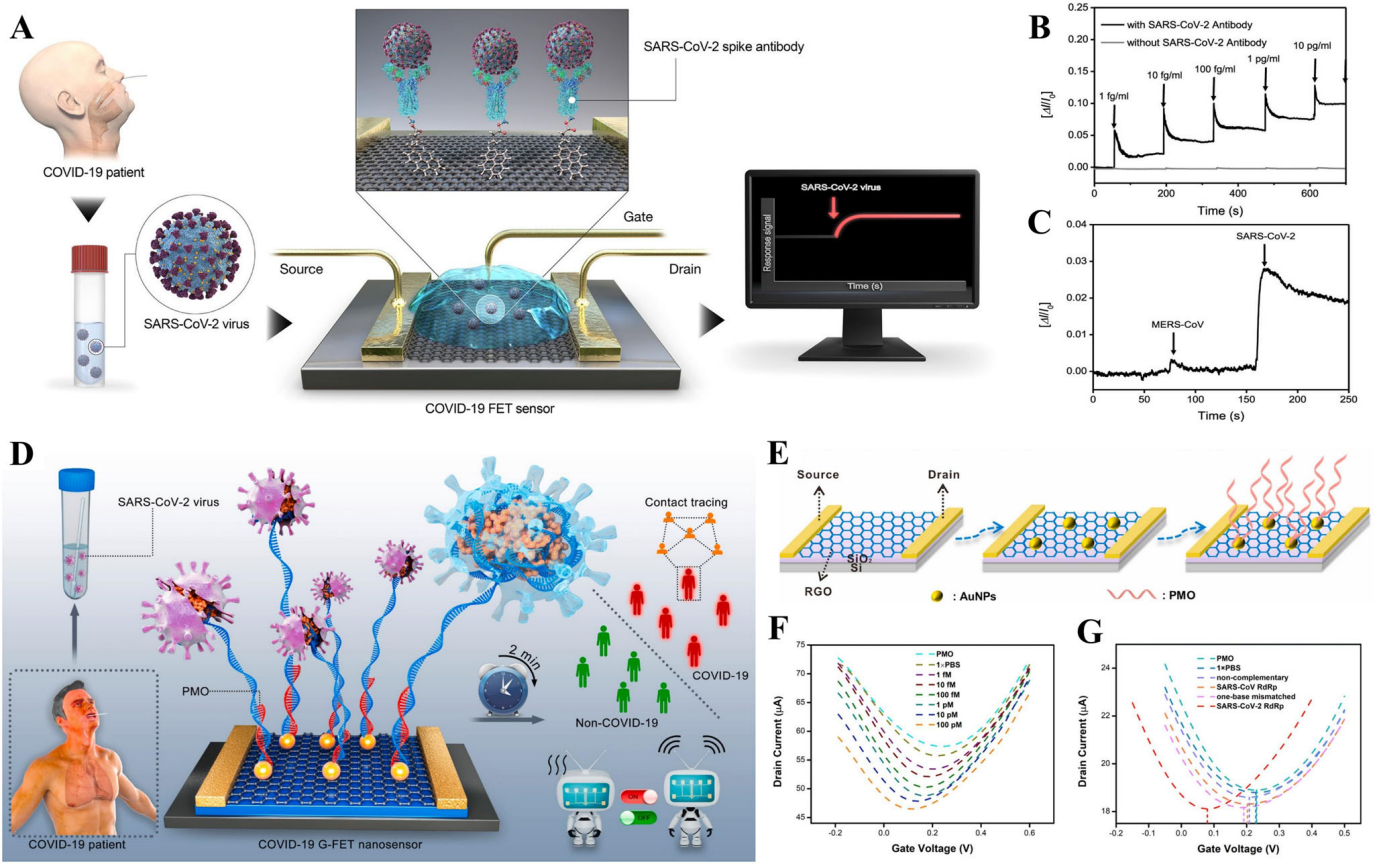
(A–C) A graphene‐based FET biosensor for detecting SARS‐CoV‐2 S protein. Reproduced with permission.^[^ ^156^ ^]^ Copyright 2020, American Chemical Society. (D–G) An AuNPs‐decorated graphene‐based PMO‐functionalized FET biosensor for detecting the SARS‐CoV‐2 RdRp gene. Reproduced with permission.^[^ ^153^ ^]^ Copyright 2021, Elsevier

In 2020, Seo et al. reported a graphene‐based FET biosensor that was modified with SARS‐CoV‐2 S antibody for detecting SARS‐CoV‐2 in clinical samples.^[^ ^156^ ^]^ The operating procedure of FET biosensor is displayed in Figure 6A.^[^ ^156^ ^]^ 1‐pyrenebutyric acid *N*‐hydroxysuccinimide ester (PBASE) was employed as the interface linker for the immobilization of SARS‐CoV‐2 S antibody on graphene. The mechanism of the biosensor can be explained as follows. When the SARS‐CoV‐2 antigen protein recognizes and forms a complex with the probe, the surface charge density changes. As a result, the potential of the gate electrode changes, which is equivalent to an external power supply. By monitoring the source‐drain channel current, the concentration of the targets can be obtained. The FET biosensor has a low LoD of 1 fg/ml in PBS for the identification of SARS‐CoV‐2 (Figure 6B).^[^ ^156^ ^]^ It also successfully detected SARS‐CoV‐2 in the culture medium (LoD of 1.6 × 10^1^ pfu/ml) and clinical specimens (LoD of 2.42 × 10^2^ copies/ml). Moreover, the biosensor can differentiate between MERS‐CoV antigen and SARS‐CoV‐2 antigen (Figure 6C),^[^ ^156^ ^]^ illustrating the specificity of the FET biosensor toward the SARS‐CoV‐2 S antigen protein. Due to its high sensitivity and specificity, this biosensor can be used to detect SARS‐CoV‐2 in clinical samples (nasopharyngeal swab) without requiring sample pretreatment and labeling like traditional methods. Thus, it holds the prospect of being employed in clinical diagnosis and decentralized PoC testing. Another group fabricated an FET biosensor by printing CNTs on an Si/SiO_2_ surface with the immobilization of SARS‐CoV‐2 S1 protein. Through noncovalent contact, a linker PBASE was used to immobilize SARS‐CoV‐2 S1 antibody on the CNT surface between the S‐D channel region. To test the electrical output of the CNT‐FET biosensor, a commercial SARS‐CoV‐2 S1 antigen was employed. The biosensor recognized the S1 antigen at concentrations ranging from 0.1 fg/ml to 5.0 pg/ml in a 10 mM AA buffer pH 6.0. The developed biosensor has an LoD of 4.12 fg/ml. The assay took only 2 to 3 min to perform.^[^ ^159^ ^]^

Li et al. demonstrated an Au NP‐decorated graphene‐FET (G‐FET) biosensor for fast and sensitive recognition of the SARS‐CoV‐2 RdRp gene, as schematically depicted in Figure 6D.^[^ ^153^ ^]^ The complementary phosphorodiamidate morpholino oligomers (PMO), an uncharged DNA analog, was functionalized on the Au NP surface as the probe (Figure 6E). The detection mechanism of the G‐FET biosensor can be described as follows. Upon binding of the PMO to the conserved sequence RdRp, a measurable Dirac point shift in the transfer characteristic curve of the G‐FET can be observed. With the RdRp gene concentration increased from 1 fM to 100 pM, a step‐by‐step left shift of the Dirac point was observed (Figure 6F).^[^ ^153^ ^]^ The specificity of the G‐FET biosensor was also verified, as shown in the comparison in transfer characteristic curves with some nonspecific sequences (Figure 3G). The G‐FET biosensor has a low LoD of 0.37 fM in PBS with a rapid response (2 min). Moreover, through direct detection of the SARS‐CoV‐2 RdRp gene, early phase infection monitoring can also be achieved. The excellent anti‐interference capability of the G‐FET biosensor has been confirmed through direct detection using undiluted samples such as throat swabs and serum. This proves that the G‐FET biosensor can be used to screen COVID‐19 patients quickly in an emergency.

**FIGURE 7:**
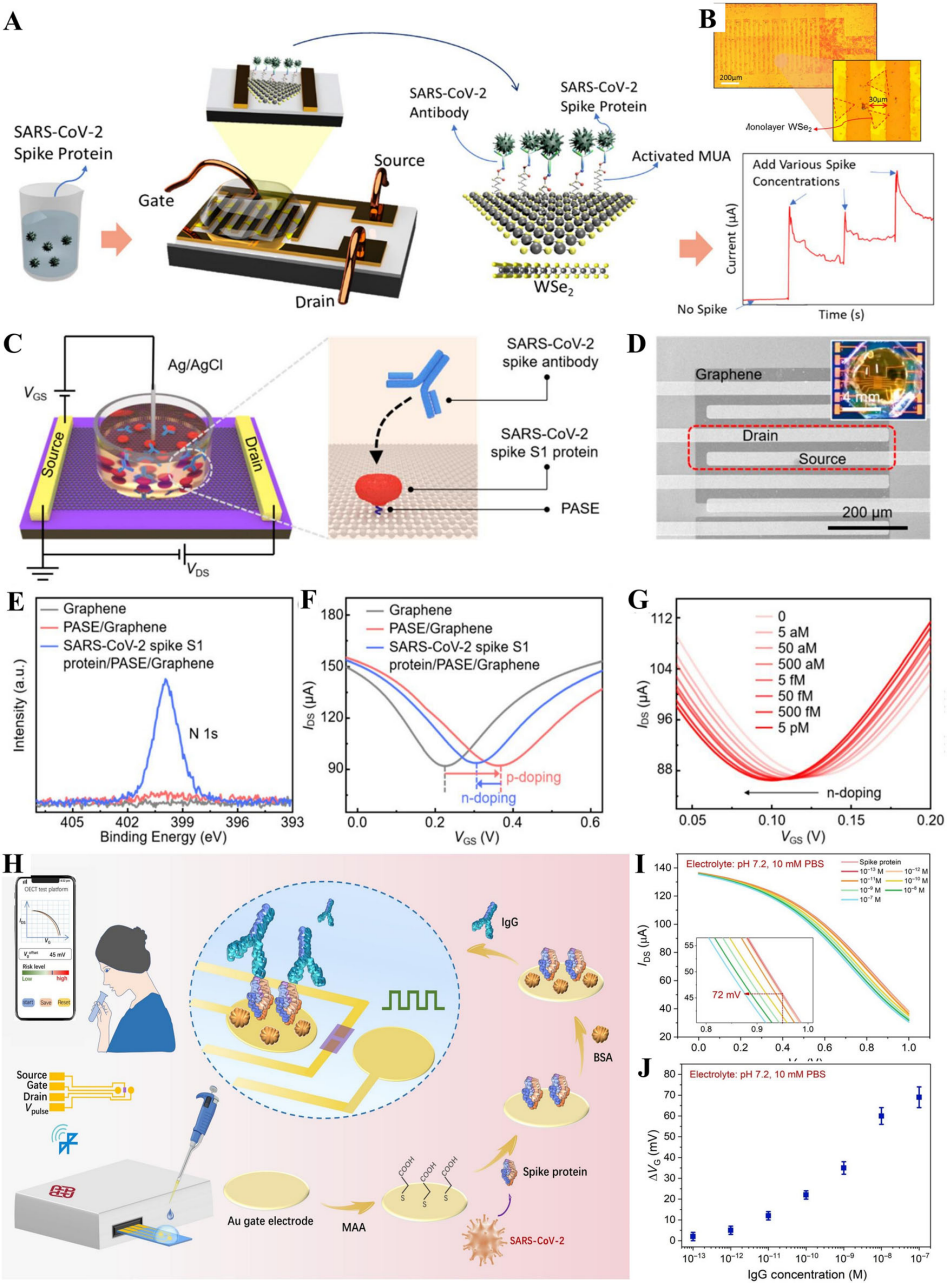
(A,B) A WSe_2_‐based FET biosensor for identification of SARS‐CoV‐2 S proteins. Reproduced with permission.^[^ ^157^ ^]^ Copyright 2021, American Chemical Society. (C–G) A liquid‐gated graphene FET (G‐FET) biosensor for detection of SARS‐CoV‐2 antibody. Reproduced with permission.^[^ ^154^ ^]^ Copyright 2021, American Chemical Society. (H–J) An organic EC transistors (OECTs)‐based biosensor for detecting SARS‐CoV‐2 IgG antibody. Reproduced with permission.^[^ ^158^ ^]^ Copyright 2021, AAAS

Fathi‐Hafshejani fabricated an FET biosensor based on monolayer tungsten diselenide (WSe_2_) for the recognition of SARS‐CoV‐2 S protein. A schematic of the fabrication and detection process of the FET biosensor is presented in Figure 7A.^[^ ^157^ ^]^ The sensing platform was constructed by functionalizing monolayer WSe_2_ crystals with SARS‐CoV‐2 monoclonal antibody using 11‐mercaptoundecanoic acid as the probe linker. Before antibody functionalization, the source and drain electrodes with interdigital structures were fabricated on monolayer WSe_2_ crystals using photolithography to increase the detection area of the FET biosensor. Figure 7B ^[^ ^157^ ^]^ illustrates the optical image of the interdigitated FET biosensor, with triangular 2D WSe_2_ crystals between the interdigitated electrodes. Effective S protein‐antibody binding was evidenced by the stepwise increase of electrical signals with increased SARS‐CoV‐2 antibody concentrations. The FET biosensor exhibits an LoD of 25 fg/μl in 0.01× PBS solution. This work demonstrates the potential of semiconducting transition metal dichalcogenide‐based FET biosensors with high sensitivity and selectivity for applications in the rapid and sensitive detection of infectious diseases.

Utilizing antigen–antibody response, SARS‐CoV‐2 proteins can also be used to identify the presence of antibodies. Kang et al. presented a G‐FET biosensor to detect SARS‐CoV‐2 antibodies (Figure 7C,D).^[^ ^154^ ^]^ The gate electrode of the liquid‐gated FET was obtained by immersing the Ag/AgCl RE in the electrolyte. The biosensor was created by immobilization of SARS‐CoV‐2 spike S1 proteins as the probes on the surface of graphene, using PBASE as the linkers. On reaction of the amine groups of SARS‐CoV‐2 spike S1 proteins with the hydroxyl‐free succinimide esters of PBASE, the spike S1 protein was modified on the graphene, as evidenced by the enhanced N 1s peaks in the X‐ray photoelectron spectroscopy image (Figure 7E). The transfer characteristic curve of the G‐FET biosensor at each modification step indicates that the S1 proteins posed an *N*‐doping effect on graphene after anchoring (Figure 7F). The special recognition of the SARS‐CoV‐2 antibody on the graphene induced an *N*‐doping effect. Therefore, carrier concentration variations in the graphene channel can cause changes in relative source‐drain current (Δ*I*_DS_/*I*_0_) as the measurable electrical response. With the increase of the concentrations of SARS‐CoV‐2 S antibody (5 aM to 5 pM), a continuous left shift in the transfer characteristic curve is observed, due to the increased *N*‐doping (Figure 7G).^[^ ^154^ ^]^ The highly sensitive G‐FET biosensor has the advantages of low LoD (2.6 aM) and rapid diagnosis (< 2 min). Clinical serum samples can be used for rapid PoC SARS‐CoV‐2 testing. It can also be employed to evaluate vaccine effectiveness, which can be achieved by continuous monitoring of the neutralizing antibody concentration after vaccination.

Liu et al. presented a SARS‐CoV‐2 IgG biosensor with an organic EC transistor (OECT). The whole detection process can be quickly completed using a portable meter, with data collected by a mobile phone. The schematic diagram of the biosensor platform and the preparation process are shown in Figure 7H.^[^ ^133^ ^]^ The OECT was fabricated on a PET substrate by photolithography, with Au source, gate, and drain electrodes achieved using magnetron sputtering. The channel between the source and drain electrodes was prepared by spin coating of poly(3,4‐ethylenedioxythiophene)–poly(styrenesulfonate) (PEDOT:PSS). The Au gate electrode was modified with biomolecules for recognition of SARS‐CoV‐2 IgG. This process involved the following steps: mercaptoacetic acid modification, binding of SARS‐CoV‐2 S protein, and BSA addition. Analyte detection was performed through three steps: addition of 5 μl of target SARS‐CoV‐2 IgG solution on the gate electrode and incubation for < 30 min, rinsing with DI water to remove IgG solution, and characterization of the device performance with a separate electrolyte. Due to the positive charge of SARS‐CoV‐2 IgG in the neutral and acidic electrolyte, an electric dipole is created on the gate electrode surface after the SARS‐CoV‐2 IgG is fixed. Thus, the surface potential of the Au gate electrode changes, inducing the effective gate voltage change. Antibody concentration can be determined by evaluating the shift of the transfer curve of the transistor before and after the antibody reaction (Figure 7I).^[^ ^133^ ^]^ The influence of SARS‐CoV‐2 IgG concentration on the relative gate voltage (Δ*V*_G_) change is depicted in Figure 7J.^[^ ^133^ ^]^ By applying voltage pulses to the OECT gate electrode during incubation to boost the movement of positively charged IgG molecules for improved antigen and antibody binding efficiency, a stable signal output can be acquired in 5 min. With the portable characteristics, OECT can achieve a quick and PoC identification of the COVID‐19 antibody. Besides, the OECT has a low LoD, which reaches 1 fM in aqueous solution and 10 fM in saliva and serum samples.

### 3.3 Plasmonic biosensors

Plasmonic biosensors utilize the properties of surface plasmons, such as the electromagnetic oscillations occurring at the metal‐dielectric interface for detecting the interaction between the ligand immobilized on the biosensor chip and the target analyte.^[^ ^125^, ^160^ ^]^ Existing plasmonic biosensors can be classified into the following categories based on different plasmonic phenomena: propagating surface plasmon resonance (SPR), localized SPR (LSPR), and surface‐enhanced infrared absorption spectroscopy (SEIRA), etc.^[^ ^160^, ^161^, ^162^, ^163^, ^164^, ^165^ ^]^ Plasmonic biosensors have the inherent label‐free advantage and can be used to detect different kinds of biomarkers including nucleic acids, proteins, and antibodies during the COVID‐19 outbreak.

**FIGURE 8:**
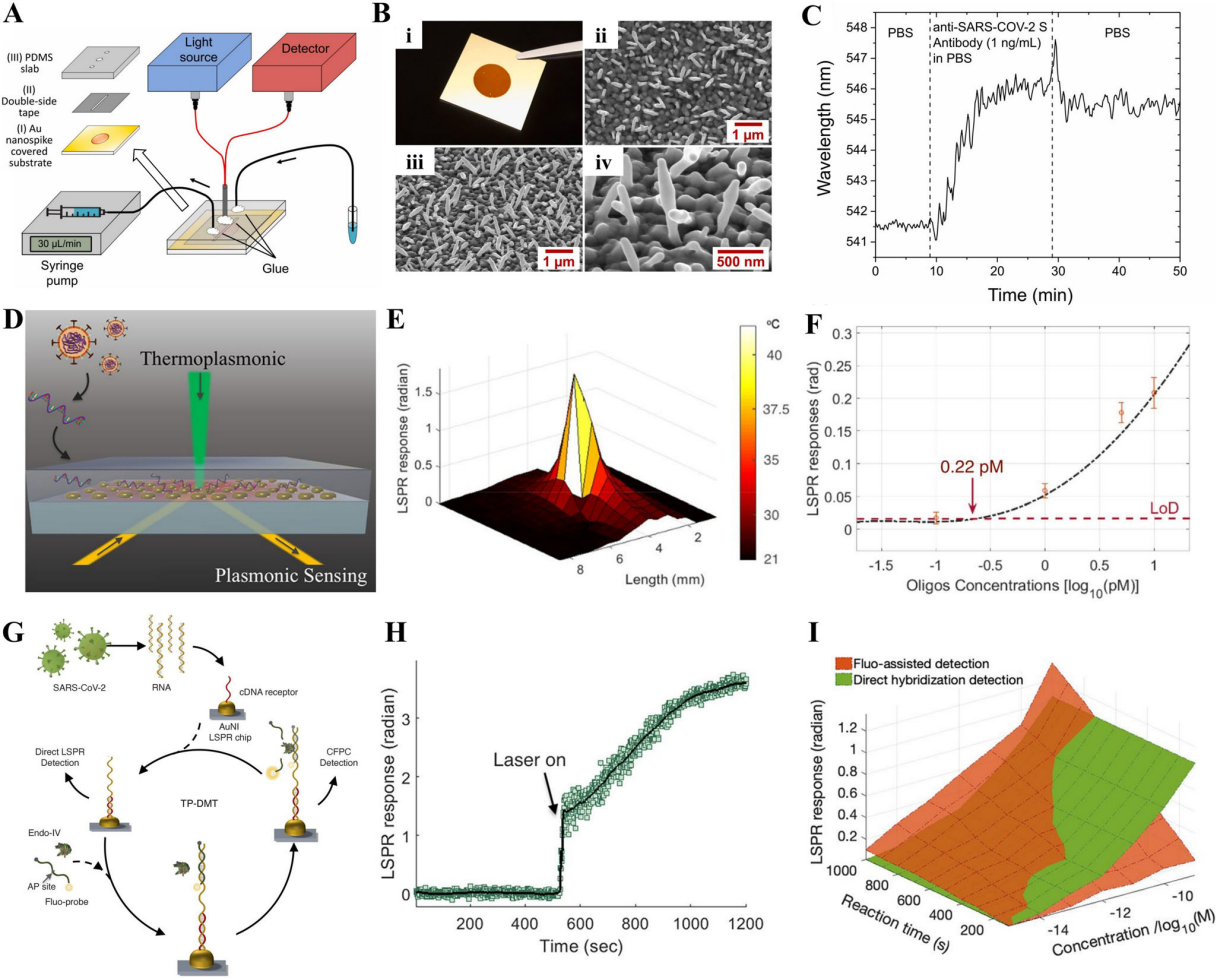
(A–C) A LSPR‐based opto‐microfluidic biosensor platform for detecting SARS‐CoV‐2 antibodies. Reproduced with permission.^[^ ^161^ ^]^ Copyright 2020, Elsevier. (D–F) A PPT enhanced LSPR‐based biosensor for identification of SARS‐CoV‐2 viral nucleic acid. Reproduced with permission.^[^ ^162^ ^]^ Copyright 2020, American Chemical Society. (G–I) A thermoplasmonic‐assisted dual‐mode sensing strategy for identification of SARS‐CoV‐2 viral RNA with high sensitivity and accuracy. Reproduced with permission.^[^ ^166^ ^]^ Copyright 2021, American Chemical Society

Funari et al. presented an opto‐microfluidic sensing platform based on the principle of LSPR for the identification of SARS‐CoV‐2 S protein antibodies. As illustrated in Figure 8A,^[^ ^161^ ^]^ the fluid samples were added to the microfluid platform using a syringe pump, while the light source on top and detector on bottom were used to deliver incident light and collect the reflected light, respectively. The opto‐microfluid platform was fabricated by electrodeposition of gold nanospike on a glass substrate, as demonstrated in the scanning electron microscope image of the Au nanospike (Figure 8B). The sensing platform showed detection of 1 ng/ml of anti‐SARS‐CoV‐2 S protein antibody in PBS with approximately 4 nm wavelength shift (Figure 8C).^[^ ^161^ ^]^ The detection mechanism is the SARS‐CoV‐2 antigen–antibody binding induced local refractive index change at the Au nanostructures interface. In consequence, a redshift of the LSPR peak was noticed, and it was in proportion to the antibody concentration. Furthermore, the opto‐microfluidic chip can detect the SARS‐CoV‐2 S protein in the sample of diluted human plasma with a low LoD of ∼0.08 ng/ml (∼0.5 pM) within 30 min. The fast and cheap fabrication procedures and its easy operation facilitate its mass production capability and accessibility to nonprofessionals. This label‐free highly sensitive and selective sensor demonstrates potential as a sensitive and fast PoC testing approach for COVID‐19 in practical application.

Qiu et al. developed a dual‐functional plasmonic photothermal (PPT) biosensor by combining the PPT effect with LSPR sensing for recognition of SARS‐CoV‐2 nucleic acid (Figure 8D).^[^ ^162^ ^]^ By integration of the PPT effect and LSPR recognition on an economical gold nanoisland (AuNI) chip, simultaneous in situ PPT heating and nucleic acid hybridization detection can be realized. Due to the different incident angles, the plasmonic resonance wavelengths of PPT and LSPR were separated from each other. This dual‐wavelength excitation method can not only improve the stability and sensitivity of the biosensor but also be used for in situ characterization of local temperature changes caused by the PPT effect. For example, the center spot at the AuNI chip rose significantly to 41°C due to the localized PPT heating (Figure 8E). Owing to the higher in situ temperature, the capability to accurately distinguish between two similar gene sequences is facilitated. On the one hand, the hybridization kinetics of complementary base pairs was improved with local PPT heating. Thus, the target virus sequence can produce a higher response per unit time, leading to rapid and sensitive detection. On the other hand, PPT heating can suppress the nonspecific bindings of mismatched sequences for improving the specificity of nucleic acid detection. The dual‐functional LSPR biosensor demonstrated high sensitivity for SARS‐CoV‐2 detection with a low LoD of 0.22 pM (Figure 8F)^[^ ^162^ ^]^ and showed prospects in clinical tests with improved accuracy.

The same research group proposed a thermoplasmonic‐assisted dual‐mode transducing (TP‐DMT) strategy by combining amplification‐free PPT enhanced LSPR sensing with amplification‐based cyclic fluorescence probe cleavage (CFPC) sensing for detecting SARS‐CoV‐2 with enhanced sensitivity and accuracy. As described in Figure 8G,^[^ ^166^ ^]^ a preliminary nonamplification detection was performed through the hybridization between SARS‐CoV‐2 viral RNA and the cDNA receptor immobilized on AuNI, with an LoD of 0.1 ± 0.04 pM. The second CPFC sensing can be achieved by the interaction between endonuclease IV and the photothermal field. Once the fluorescent DNA probe hybridizes with the target viral sequence, the endonuclease IV cleaves the AP site of the probe to cut it into two short strands. Double‐helix dissociation of the shortened fluorescent probe is induced under the PPT heating. At this moment, the phase change of LSPR near AuNI is excited, expressed by an instantaneous phase jump in Figure 8H.^[^ ^166^ ^]^ The cyclically released fluorescent probes can induce the instantaneous and cumulative LSPR response, thereby lowering the LoD to 0.275 ± 0.051 fM. As depicted in Figure 8I,^[^ ^166^ ^]^ when clinical samples have a lower viral load (<0.1 pM), the CFPC sensing outranges direct LSPR sensing in its accuracy. Overall, the combined TP‐DMT strategy has self‐validating properties for a more reliable testing result. Even for clinical samples containing trace amounts of SARS‐CoV‐2 viral sequences, the TP‐DMT‐based biosensor shows advantages of low LoD and rapid sensing (within 30 min).

**FIGURE 9:**
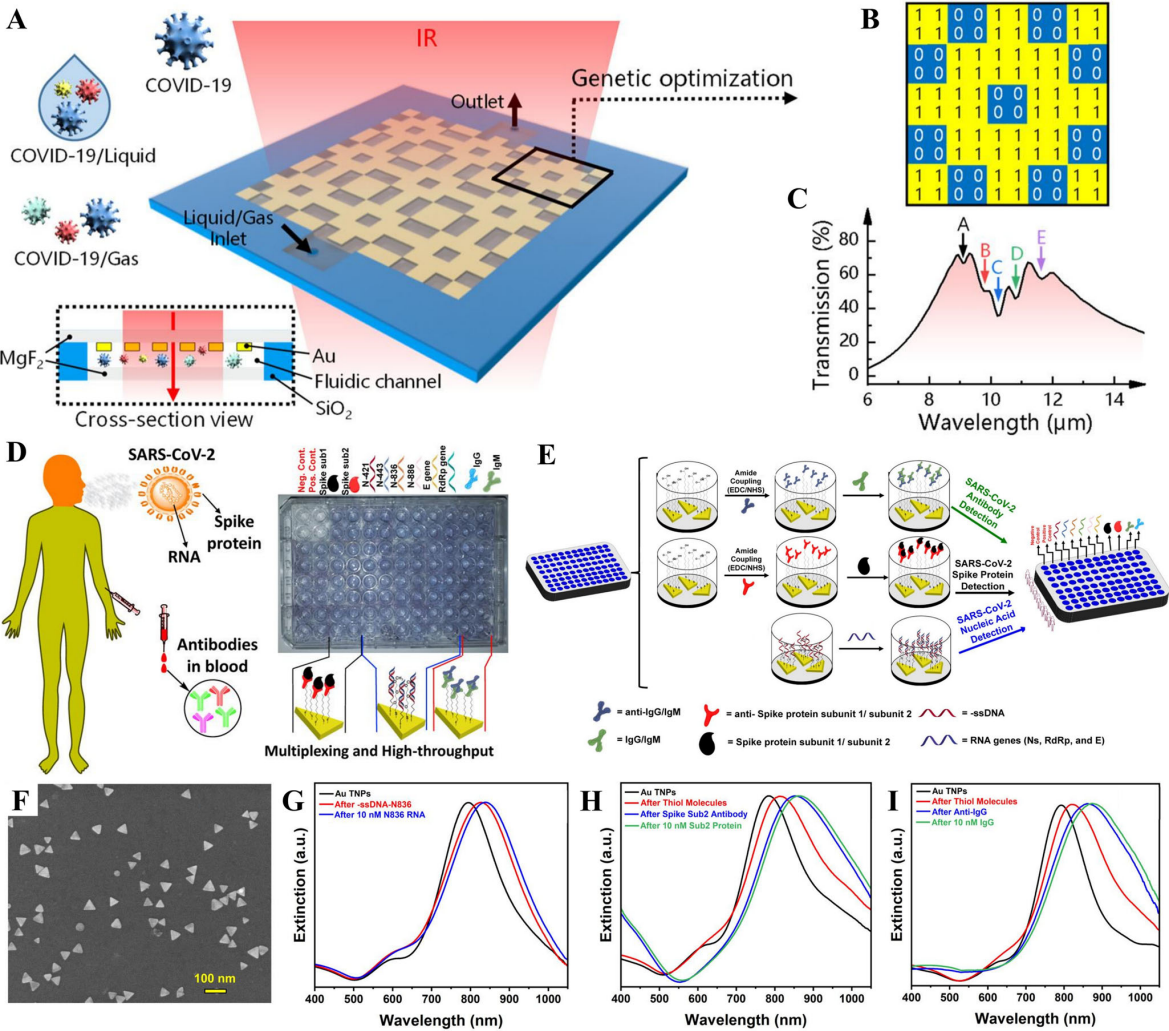
(A–C) A SEIRA plasmonic biosensor enhanced with genetic algorithm program for detecting SARS‐CoV‐2 virus. Adapted with permission.^[^ ^167^ ^]^ Copyright 2021, American Chemical Society. (D–I) A label‐free nanoplasmonic‐based biosensor platform for multiplexed and high throughput recognition of 10 biomarkers (RNA/S protein/IgG/IgM) of SARS‐CoV‐2. Adapted with permission.^[^ ^163^ ^]^ Copyright 2021, American Chemical Society

As reported by Li et al., SEIRA plasmonic biosensor augmented by a genetic algorithm (GA) was developed for on‐site COVID‐19 diagnosis. As illustrated in Figure 9A,^[^ ^167^ ^]^ the Au nanostructure was deposited on a magnesium fluoride (MgF_2_) substrate and a microfluidic channel was formed between another MgF_2_ substrate. The infrared light passed successively through the top MgF_2_, Au nanostructure, microfluidic channel, and MgF_2_ at the bottom and was subsequently collected by an infrared detector. The incident light induced the plasmon resonance on the surface of Au nanostructure, which was coupled with the COVID‐19 molecular vibration and led to a change in the transmission spectrum. For enhancement of the sensitivity for SARS‐CoV‐2 detection, GA was used for the optimization of the structural design of metal nanostructures (Figure 9B). Five obvious vibration peaks were observed owing to the coupling of virus molecules and irradiation‐induced plasmon resonance (Figure 9C).^[^ ^167^ ^]^ The plasmonic biosensor can be employed to recognize the SARS‐CoV‐2 virus in liquid (nasopharyngeal swabs/saliva) and gaseous (exhaled breath) samples. Rapid quantitative detection of SARS‐CoV‐2 with a high sensitivity of 1.66%/nm was achieved in a liquid environment. Moreover, the GA enhanced biosensor can be used for mutant virus detection due to its characteristics of infrared fingerprint recognition. This work also provided a rapid approach for early pre‐screening of COVID‐19.

Masterson et al. developed a label‐free nanoplasmonic biosensor for COVID‐19 diagnosis via the detection of 10 different biomarkers. The biosensor can achieve multiplexed and high‐throughput recognition of RNA gene sequences, SARS‐CoV‐2 S protein, and plasma antibodies in one test run (Figure 9D).^[^ ^163^ ^]^ A schematic of the fabrication procedure is presented in Figure 9E. Specifically, the biosensor was constructed by chemically modifying gold triangular nanoprisms (Au TNPs) onto glass coverslips (Figure 9F).^[^ ^163^ ^]^ The glass coverslips were subsequently glued to the bottom of a 96‐well plate. For the construction of biosensors intended for different target analytes, such as SARS‐CoV‐2 RNA, spike protein, or IgG and IgM antibodies, different receptors were functionalized onto the Au TNPs. The mechanism of the biosensor can be described as follows. Target analyte adsorption produces a local refractive index change based on LSPR, which can be reflected as the shift of λLSPR position in UV–vis extinction spectra (Figure 9G–I).^[^ ^163^ ^]^ The multiplexed and high throughput biosensor can recognize SARS‐CoV‐2 with high sensitivity [low LoD (89 aM)] as well as high specificity throughout infection. The screening test demonstrated a calculated sensitivity of 100% (80/80) and a specificity > 96% (77/80), with a positive predictive value of 98% and a negative predictive value of 100% at 5% prevalence.

### 3.4 Colorimetric biosensors

Colorimetric biosensors indicate the presence of target biomarkers via a change in the color of the solution.^[^ ^168^, ^169^, ^170^, ^171^ ^]^ Generally, the specific binding between functionalized NPs and target biomarkers leads to changes in NP size and distance, which is visualized as the change in solution color. Colorimetric sensors in the visible light range generate an observable signal, which can be perceived with naked eyes. Target biomarkers can also be quantified based on the visible color change using simple optical devices like smartphones.^[^ ^168^, ^170^, ^171^ ^]^ Back in 1997, Prof. Chad A. Mirkin had reported a colorimetric biosensor utilizing the aggregation of Au NPs.^[^ ^172^ ^]^ Au NPs are modified with oligonucleotides as probes. When the target DNA is present, the probes hybridize to form a polymeric network of NPs. This causes the color of the solution to change from red to blue.

**FIGURE 10:**
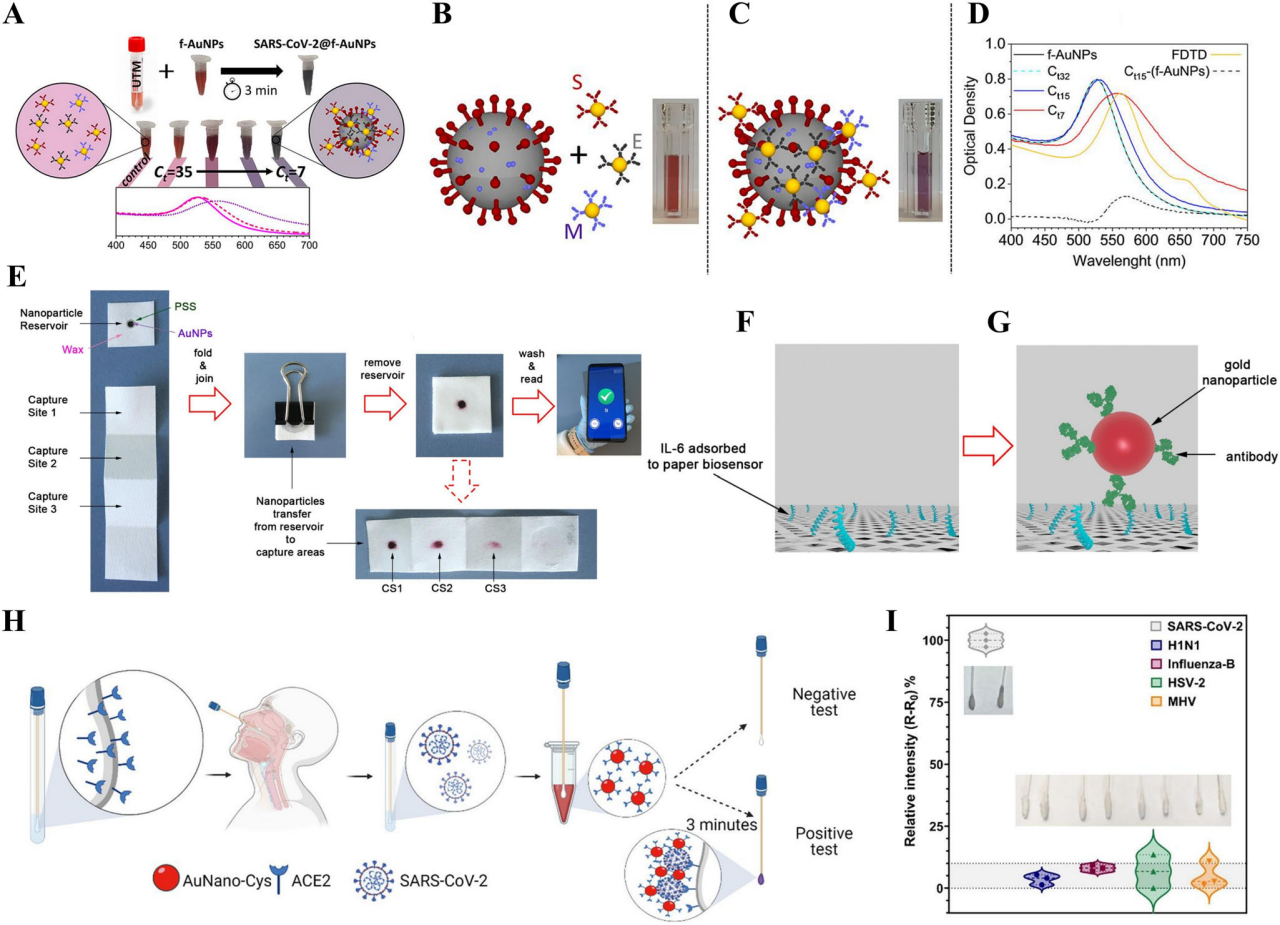
(A–D) A colorimetric biosensor with AuNP for detecting SARS‐CoV‐2 surface proteins. Adapted with permission.^[^ ^169^ ^]^ Copyright 2020, American Chemical Society. (E–G) A colorimetric biosensor with folded paper design for detection of SARS‐CoV‐2 via recognition of cytokine IL‐6 with enhanced sensitivity. Adapted with permission.^[^ ^168^ ^]^ Copyright 2021, Elsevier. (H,I) A low‐cost colorimetric biosensor using cotton swabs for minute‐scale SARS‐CoV‐2 detection. Adapted with permission.^[^ ^174^ ^]^ Copyright 2021, American Chemical Society

Ventura et al. developed a colorimetric biosensor using Au NPs for detecting SARS‐CoV‐2 in nasal and throat swabs (Figure 10A).^[^ ^169^ ^]^ The Au NPs were functionalized with S, E, and M antibodies targeting the three SARS‐CoV‐2 surface proteins (spike, envelope, and membrane, respectively) using photochemical immobilization technique. A pink colloidal solution was obtained with the anti‐SARS‐CoV‐2 functionalized Au NPs (f‐Au NPs), as depicted in Figure 10B. Upon mixing of the f‐Au NP colloidal solution with SARS‐CoV‐2 solution, the protein‐antibody binding caused a layer of NPs to form on the surface of SARS‐CoV‐2. Consequently, the resonance peak was red‐shifted in the extinction spectrum, which can also be reflected as the optical color variation of the solution from pink to purple (Figure 10C,D).^[^ ^169^ ^]^ The colorimetric biosensor demonstrated high sensitivity (96%) and specificity (96%) when analyzing 94 real samples with 45 positives [threshold cycle (C_t_) ≤ 35] and 49 negatives (*C*_t_ > 35) as confirmed by standard PCR test. Notably, viral loads at *C*_t_ = 36.5 can be identified by the colorimetric biosensor, which is comparable to the detection limit of the quantitative PCR test. The colorimetric biosensor did not require any pretreatment since it utilized the virion instead of RNA. This sensing technique with a single step shows potential for PoC testing and large‐scale screening of COVID‐19.

Adrover‐Jaume et al. reported a folded paper‐based colorimetric biosensor for rapid and sensitive detection of IL‐6 levels in both blood and respiratory samples. As a type of lymphokine produced by activated T cells and fibroblasts, IL‐6 levels in serum have been successfully employed for predicting the disease progression in COVID‐19 patients.^[^ ^173^ ^]^ As shown in Figure 10E,^[^ ^168^ ^]^ the biosensor comprised two parts. The first part was a reservoir paper containing anti‐IL‐6 antibody modified Au NPs. The second part was a paper strip having three capture sites (CSs) for adding sample solution. After the addition of a drop of sample solution to each CS, the paper strip was folded, and the reservoir was pressed on its top for 5 min. In this process, the antibody coated Au NPs transferred perpendicularly from the reservoir to three CSs. The specific interaction between antibodies immobilized on Au NPs and IL‐6 cytokine (Figure 10F,G)^[^ ^168^ ^]^ induced a colorimetric signal collected by a smartphone. The colored point pixel intensity strongly correlated with IL‐6 concentration in the sample. By incorporating the colorimetric signals in three colored spots, the signals were amplified to achieve an LoD of 10^−3^ pg/ml in PBS solution in a wide detection range of 10^−3^–10^2^ pg/ml. This low LoD allowed IL‐6 detection in the diluted blood sample at 1.3 pg/ml within 10 min. The properties of rapid testing with different kinds of samples (blood and respiratory samples) ensured the capabilities of decentralized COVID‐19 diagnosis at home.

Ferreira et al. presented a colorimetric biosensor called COVID‐19 Low‐cost Optodiagnostic for Rapid testing (COLOR) using cotton swabs and ACE2 modified Au NPs for direct SARS‐CoV‐2 detection. As illustrated in Figure 10H,^[^ ^174^ ^]^ the testing procedures involved the following steps: ACE2 immobilization on the cotton swabs, immersion and incubation of the cotton swabs into the clinical SARS‐CoV‐2 samples for the binding of ACE2 with virus S protein, and incubation of the as‐obtained cotton swabs into Au NPs‐cysteamine‐ACE2 solution for 3 min. For positive patients, the solution color changed from red to purple because of the cotton‐ACE2/SARS‐CoV‐2/Au NPs‐cysteamine‐ACE2 aggregation. With RGB software on smartphones, the color change can be quantified as relative color intensity values for analysis. The COLOR can detect SARS‐CoV‐2 S protein with a low LoD of 0.154 pg/ml. Cross‐reactivity studies were also performed to verify the high selectivity of COLOR for SARS‐CoV‐2 (Figure 10I).^[^ ^174^ ^]^ Through the testing of 100 nasopharyngeal/oropharyngeal samples, its high sensitivity (96%), specificity (84%), and accuracy (90%) were also confirmed. Another significant advantage of the COLOR biosensor is the low cost. Each COLOR test costs as low as 15 cents, while providing a simple, rapid (within 5 min), accurate, and high‐frequency‐capable COVID‐19 test.

**FIGURE 11:**
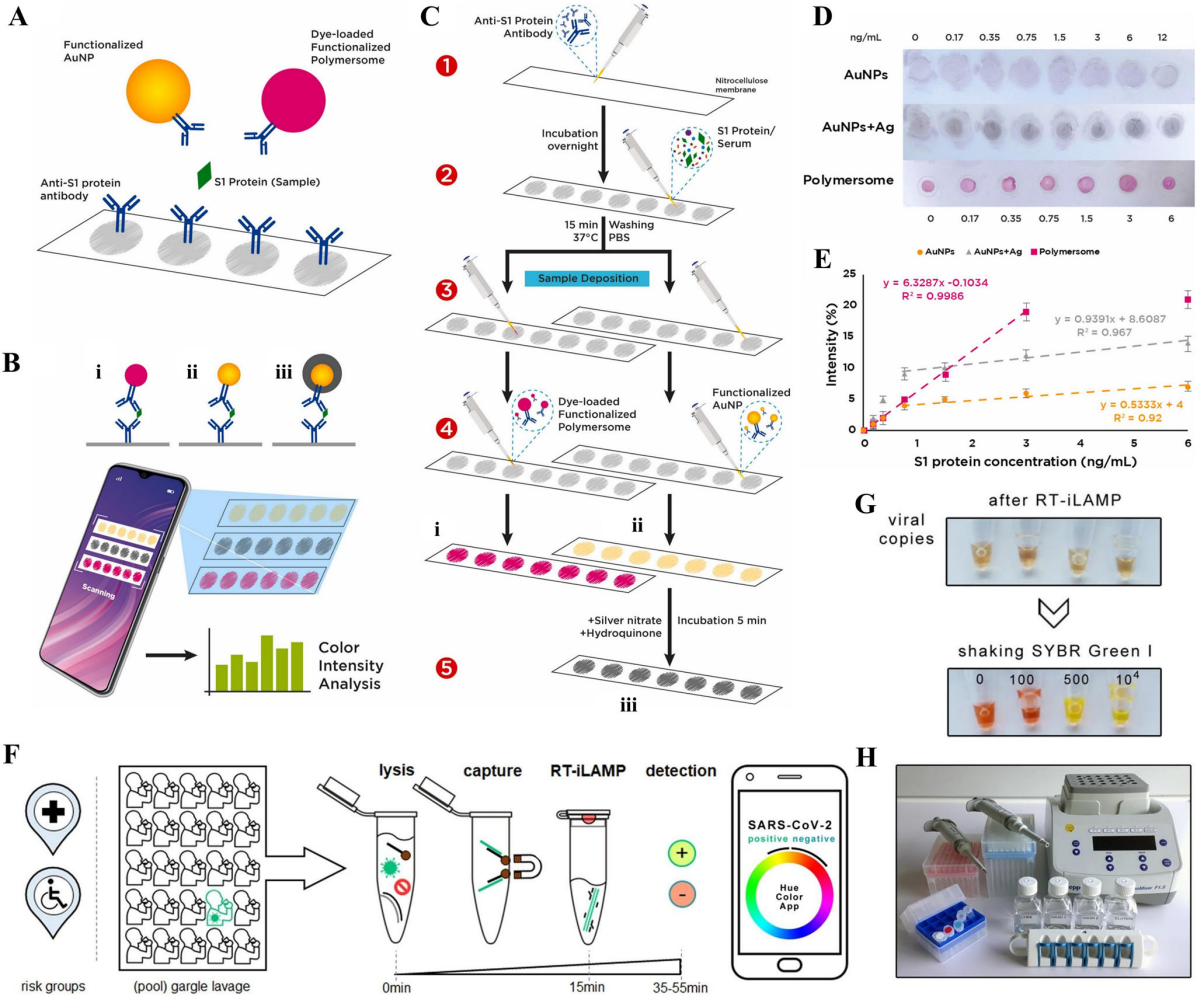
(A–E) A colorimetric paper‐based dot blot biosensor using fuchsine dye‐loaded polymersome for detecting SARS‐CoV‐2 S protein. Adapted with permission.^[^ ^170^ ^]^ Copyright 2021, Elsevier. (F–H) A colorimetric LAMP assay assisted with smartphone color capturing for detecting SARS‐CoV‐2 RNA. Adapted with permission.^[^ ^171^ ^]^ Copyright 2021, Springer Nature

Also assisted with a smartphone for color image analysis, Ghorbanizamani et al. reported a colorimetric paper‐based dot blot S protein biosensor for the detection of SARS‐CoV‐2 S protein (Figure 11A).^[^ ^170^ ^]^ Polymersome loaded with fuchsine dye and functionalized with SARS‐CoV‐2 S protein antibodies was used to improve the sensitivity and stability of the sensor. Parallel experiments using Au NPs and silver enhanced Au NPs (Au‐NPs‐Ag)‐based colorimetric assays were designed to evidence the above features (Figure 11B). As shown in Figure 11C, for the construction of the paper‐based colorimetric biosensor, anti‐S1 protein antibodies were first modified onto the surface of a nitrocellulose membrane. Test samples were subsequently deposited by adding human serums. Finally, the functionalized NPs were added for colorimetric revelation. The fuchsine dye‐loaded polymersome demonstrated a higher color intensity compared with Au NPs and Au‐NPs‐Ag, as observed from the visual views of the dot blots (Figure 11D). More intuitively, the color intensities of the dot blots with fuchsine dye‐loaded polymersome showed higher sensitivity and linearity, as depicted in Figure 11E ^[^ ^170^ ^]^ showing the relationship between color intensity and target analyte concentration. This smartphone‐assisted colorimetric biosensor facilitates PoC detection of SARS‐CoV‐2 with the merits of timely testing and fast results.

Bokelmann et al. reported a facile capture and improved LAMP technique for the identification of SARS‐CoV‐2 RNA by combining hybrid capture‐based RNA extraction with colorimetric reverse transcription (RT)‐LAMP assay. To reduce human error in the reading process, a mobile application was developed to obtain the color hue value. The test procedure is illustrated in Figure 11F.^[^ ^171^ ^]^ Twenty‐six gargle lavage samples were collected from the COVID‐19 risk groups, which was followed by combined lysis to release and capture the nucleic acid. Obtained RNA was subjected to RT‐LAMP for 25–30 min, while maintaining the temperature at 65°C. Color variation was generated by adding a drop of SYBR green I dye in the tube and shaking (Figure 11G). SARS‐CoV‐2 can be visually detected by monitoring the color change using a free “camera color picker” smartphone application. The apparent color change can be quantitatively converted to a single numerical hue value for precise detection. Since only a small amount of reagents and instruments are demanded (Figure 11H),^[^ ^171^ ^]^ this approach integrating nucleic acid extraction, LAMP, and automatic colorimetric data reading provides a way of rapid (<1 h) and inexpensive (∼1 Euro) PoC testing for SARS‐CoV‐2 identification.

### 3.5 Mass spectrometry methods

Mass spectrometry (MS) is a crucial analytical strategy for genomics, proteomics, metabolomics, and human disease microflora studies owing to its unique features such as sensitivity, specificity, and rapidity.^[^ ^175^ ^]^ MS techniques have successfully identified and quantified specific subsets of proteins/peptides at the resolution of separate atomic‐level amino acids with excellent sensitivity and repeatability. Thus, MS techniques exhibit a speedy, reliable, and economical alternative for COVID‐19 detection from oral and nasopharyngeal swabs.^[^ ^176^, ^177^, ^178^ ^]^ Multiple reaction monitoring can identify two specific peptides of COVID‐19 per test with 100% selectivity and 90.5% sensitivity versus RT‐PCR. Notably, these peptides could be identified in infected people who have recovered and tested negative for COVID‐19 by RT‐PCR. Hence, the MS technique is in spotlight owing to its sensitivity and capability in the timely diagnosis of symptomatic and asymptomatic cases in a 2.3 min gradient run.^[^ ^177^ ^]^ Moreover, MS techniques can be integrated with other technologies such as gas chromatography, liquid chromatography (LC), inductively coupled plasma (ICP), and matrix‐assisted laser desorption/ionization (MALDI) to boost sensitivity, specificity, analysis speed, and LoD. Therefore, tandem MS is a promising strategy as a reliable diagnostic apparatus in the premature COVID‐19 pandemic.

A proof‐of‐concept study demonstrated that tandem MS can be utilized to identify SARS‐CoV‐2 directly in the urine. A subset of urine samples including those from COVID‐19 infected and noninfected individuals, analyzed by the tandem MS, was applied for analyzing proteomics quantitatively.^[^ ^179^ ^]^ Proteins in positive urine samples underwent the enrichment process in the acute phase response, immune response, and the complement system. Remarkably, diverse renal proteins, including amino acid transporter (SLC36A2), podocin (NPHS2), and sodium/glucose cotransporter 5 (SLC5A10), concerned with normal kidney function, were found to be reduced in the urine of infected people. In general, the identification of viral antigens by LC‐MS/MS as well as the urinary proteome diversity may help researchers better understand COVID‐19 etiology. The latest version of LC‐MS‐based methods is capable of directly identifying low quantities of viral proteins by integrating field asymmetric ion mobility spectroscopy (FAIMS)‐parallel reaction monitoring (PRM) with LC‐MS/MS.^[^ ^180^ ^]^ The integration of FAIMS significantly enhances the sensitivity of the tandem MS due to orthogonal gas‐phase separation that rapidly separates charged ions based on their size; hence, the integrated tandem MS is a potential candidate for clinical diagnostics. To demonstrate the performance of FAIMS‐integrated LC‐MS/MS, N protein was selected as the target antigen in various specimens, including purified virus, recombinant viral protein, and nasopharyngeal swab specimens from infected cases. Consequently, this system exhibited 97.8% sensitivity and 100% specificity with a high output accuracy of 100 samples per day, which could be employed on other sample types such as saliva and urine. Additionally, an ultra‐high‐performance LC‐MS/MS can diagnose COVID‐19 based on vitamin C serum levels.^[^ ^181^ ^]^ The patients typically have a low concentration of vitamin C (2.00 mg/L), roughly 5‐fold lower than healthy people (9.23 mg/L). To elucidate the capabilities of single particle‐inductively coupled plasma‐tandem MS (SP‐ICP‐MS/MS) and homogeneous analysis, SP‐ICP‐MS/MS was devised using Au and Ag NP‐based NA probes for the simultaneous detection of COVID‐19 and influenza (H3N2). The corresponding probes (Au NP or Ag NP) become bigger aggregates in the presence of specific COVID‐19 and H3N2 nucleic acids, leading to enhanced pulse signal intensity and reduced pulse signal frequency owing to no interference of Au and Ag probes as well as no need for separation or washing procedures. Both Au and Ag probes enable easy operation, reduced analysis time, and enhanced analysis efficiency.^[^ ^182^ ^]^ Consequently, the SP‐ICP‐MS/MS exhibited a linear range of 5–1000 pmol/L with a low LoD of target nucleic acid. By applying various modification sequences on NP probes, the proposed SP‐ICP‐MS/MS approach can potentially identify nucleic acids, proteins, cells, and other biological analytes. Another high‐throughput anchored proteomic analysis was developed to squarely uncover nucleoprotein peptides of COVID‐19, such as DGI, IGM, HSG, and SYE, from nasopharyngeal and oropharyngeal swabs.^[^ ^178^ ^]^ Ninety‐six samples were automatically collected within 4 h by deploying the modified magnetic particle‐based protein analysis on a robotic liquid handler. Four‐channel turbulent flow chromatography (TFC) integrated with triple quadrupole MS accomplishes high‐volume testing much better than LC‐MS/MS. The TFC/MS technique can analyze four samples simultaneously in <10 min, enabling testing of >500 samples per day.

MALDI‐time of flight MS (MALDI‐TOF MS) has attracted attention as a viable strategy for microbial detection and diagnosis in recent years. In 2021, Rybicka et al. proved that MALDI‐TOF MS outperformed RT‐PCR in the identification of COVID‐19 RNA.^[^ ^183^ ^]^ The study included 168 participants with suspected respiratory illness. Seventeen samples exhibited contradictory findings on analysis by RT‐PCR and MS simultaneously (10.12%). The MS‐based test confirmed that 13 out of the 15 samples were publicly determined as presumptive positive. Additionally, at least one out of four samples that were declared as negative by the official RT‐PCR was found to be positive by MS assay. The MS‐based assay manifests outstanding sensitivity in detecting COVID‐19; thus, MALDI‐TOF MS has potential for detecting and discriminating variants within the genome of COVID‐19. Saliva specimens are considered as a representative and potential approach for rapid and safe sampling in a large variety of populations. However, techniques for COVID‐19 detection in saliva are not well known and limited. To surpass this barrier, Hernandez et al. conducted and verified RT‐PCR/MALDI‐TOF MS‐based assay, which is considered Agena MassARRAY^®^ now.^[^ ^184^ ^]^ The analytical sensitivity and specificity of the platform were appraised and determined with an LoD of 1562.5 copies/ml.

**FIGURE 12:**
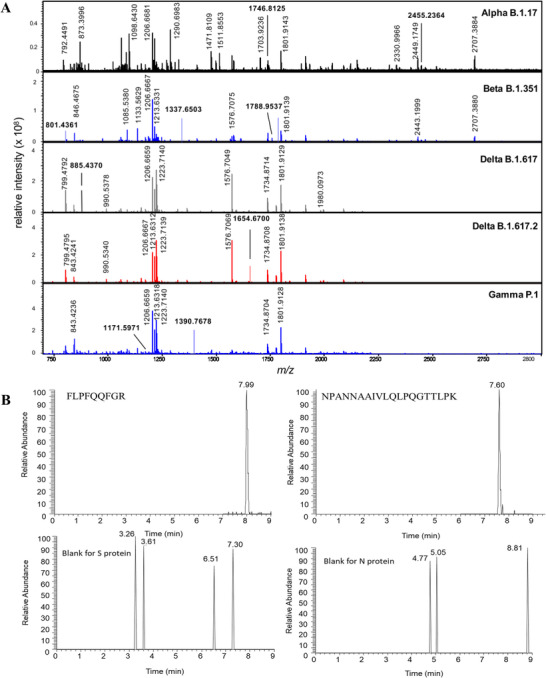
(A) MALDI/FT/ICR/MS mass spectra for the doubly‐digested (trypsin + GluC) recombinant S‐protein for five major variants of COVID‐19. Adapted with permission.^[^ ^185^ ^]^ Copyright 2021, Springer Nature. (B) The extracted ion chromatograms (XIC) for the quantifier ions. Left: The XIC for the quantifier ion of S and N proteins at LoD (up) and blank matrices (bottom). Adapted with permission.^[^ ^186^ ^]^ Copyright 2021, Elsevier

SARS‐CoV‐2 mutant strains were identified and quickly distinguished by mass mapping employed by high‐resolution MS across five important mutants. Deletions or mutations on the S protein surface cause variation in the mass maps of variants, including Alpha, Beta, Gamma, and Delta. As a consequence, peptides with distinct masses are specific for their corresponding variants.^[^ ^185^ ^]^ To detect and understand viral evolution, the same mass map profiles have been used to build phylogenetic trees without the requirement of protein (or gene) sequence or alignment. The integrated solutions outperform traditional gene‐based approaches by combining the ease of generating protein mass maps with the efficiency and accuracy of spectrometric analysis. As anticipated, Fourier‐transform ion cyclotron resonance MS coupled with MALDI exhibits many common ions, such as those at mass‐to‐charge ratio (*m*/*z*) 1206 and 1801, which are the consequences of a missing cleaved peptide including residues 517–228 and 341–355, respectively. Notably, the actual numbering of these residues and other peptide segments differs from the original strain due to the introduction of deletion sites in some variants. For instance, three peptides at *m*/*z* 801.4361, 1337.6503, and 1788.9537, representing residues 238–246, 472–484, and 238–253 corresponding to the 242–244 deletion, E484K and R246I mutations, identify the Beta variants. The Delta variants are differentiated from variants of concern, as well as from each other, in which T95I and R158G mutations were located at *m*/*z* 885.4370 and 1654.6700, as shown in Figure 12A.^[^ ^185^ ^]^

COVID‐19 vaccines are the most hopeful strategy to tackle the COVID‐19 outburst. There is an urgent need to ensure quality control and provide robust analytical methods to assess vaccine efficacy and safety over time. In vivo animal testing, namely serum‐based ELISA, has been widely utilized for vaccine quality control. These procedures, which are inaccurate and time‐consuming, involve expensive availability and specific antibodies. Hence, developing robust bioanalytical assays as an alternative is of great interest for evaluating complex vaccination antigens qualitatively and quantitatively. To simultaneously detect S and N proteins, a PRM‐based LC‐MS/MS approach was devised employing optimized unique peptides. Five copies of N and S proteins mixed at different concentrations demonstrated excellent linearity and repeatability. Among them, the most sensitive peptides of both proteins at the LoD of 0.1 μg/ml were observed, as shown in Figure 12B.^[^ ^186^ ^]^ This system exhibited ultra‐high selectivity toward both proteins due to no endogenous interference appearing in blank samples. Moreover, S and N proteins were quantified in 9 batches of inactivated vaccines wherein their concentration did not change. Therefore, this approach is convincing in assessing the quality of other S and/or N protein‐based COVID‐19 vaccines as well as other viral vector and protein subunit‐based vaccines. An isotope dilution MS (IDMS) approach possesses all of the required characteristics for reliable quantification in SARS‐CoV‐2 protein‐based vaccines and as goals for quick diagnostic testing.^[^ ^187^ ^]^ Quantifying and averaging five peptides for S protein (three peptides in the S1 and two peptides in the S2) and four peptides for N protein resulted in absolute quantification. The total relative standard deviation for S and N proteins was 3.67% and 5.11%, respectively. For the measurement of specific S and N proteins, the IDMS provides speed (5 h for total analytical time), sensitivity (LOQ: 10 fmol/L), and accuracy. IDMS is being applied in the manufacturing of vaccines under FDA approval owing to its quick, accurate, and reliable protein quantification.

## 4 CONCLUSIONS AND PERSPECTIVES

Considering the current trend of the COVID‐19 pandemic, in addition to protective measures such as face masks and social distancing, we should also take proactive approaches such as PoC COVID‐19 testing to prevent transmission by asymptomatic and early‐stage patients.^[^ ^130^, ^188^, ^189^ ^]^ Although the widely recognized PCR method has high accuracy and specificity, its use as a PoC testing method is hindered owing to the expensive equipment and long detection period.^[^ ^129^ ^]^ In comparison, biosensors provide an alternative solution for COVID‐19 detection with advantages of low cost and fast detection, enabling control of virus spread.^[^ ^126^ ^]^ Biosensors normally do not require a professional personnel to operate, which means a PoC test or even real‐time monitoring can be conducted timely by patients themselves. In this way, cluster infections possibly caused by visiting suspected patients to the hospital or other institutions for testing can be avoided. Widespread use of biosensors will have a significant effect on the early diagnosis and control of COVID‐19 and also aid in reducing pressure on the healthcare system.^[^ ^130^ ^]^

In this review, biosensors based on some promising mechanisms for the detection of COVID‐19 have been discussed at length. To date, various nanomaterials such as Au NPs,^[^ ^116^, ^132^, ^161^, ^162^, ^163^ ^]^ conducting polymers,^[^ ^37^ ^]^ graphene,^[^ ^154^, ^156^ ^]^ WSe_2_,^[^ ^157^ ^]^ and metal oxide^[^ ^30^ ^]^ have been used to create biosensors with enhanced SARS‐CoV‐2 detection capabilities. This is because of their excellent properties of high specific surface area, multiple reaction sites, and optical transparency. With these nanomaterials, biosensors based on various transduction mechanisms have been developed, such as EC,^[^ ^37^, ^54^, ^55^, ^134^, ^135^, ^137^, ^190^ ^]^ FET,^[^ ^153^, ^154^, ^156^, ^157^, ^158^ ^]^ plasmonic,^[^ ^161^, ^162^, ^163^, ^166^, ^167^ ^]^ and colorimetric biosensors.^[^ ^168^, ^169^, ^170^, ^171^, ^174^ ^]^ Furthermore, diverse analytes can be employed for SARS‐CoV‐2 detection, including viral RNA, S protein, antibodies, and lymphokine. To date, biosensors have achieved fast pre‐screening, on‐site diagnosis, and on‐body monitoring of COVID‐19 with high sensitivity, accuracy, and specificity. Some of them demonstrate potential for massive testing due to their ease of operation, low cost, and rapid test result.^[^ ^154^, ^161^, ^174^ ^]^ In addition, by using applications on mobile phones, on‐site imaging and analysis have been realized for quantitative COVID‐19 detection.^[^ ^168^, ^170^, ^171^, ^174^ ^]^ Although biosensors for COVID‐19 detection have the characteristics of fast detection and low cost, the occurrence of false‐negative cases due to the variation in viral content at different stages of infection, differences in sampling methods, and the existence of interferents should also be considered. Besides, the detection sensitivity and accuracy of biosensors are lower than those of PCR and ELISA methods. Therefore, they are recommended to be used for qualitative detection and cannot be used as the basis for COVID‐19 diagnosis. However, with the continuous development of biosensors with improved sensitivity and accuracy, biosensors may play a role in rapid large‐scale screening and areas with scarce medical resources. Without a doubt, they can also be used complementary to traditional PCR methods.

Despite the current rapid progress of biosensors based on nanomaterials for COVID‐19 diagnosis, further work needs to be done to establish a quantitative standard to compare biosensors with different mechanisms. Besides, the reliability and reproducibility of biosensors should be studied in detail to guarantee more stable and consistent testing results and mass producibility. Moreover, testing criteria for samples from diverse sources (nasopharyngeal swab, sputum saliva, exhaled breath, and feces) should be standardized to fulfill different needs and testify to each other for improved accuracy. In the future, with the aid of the Internet of Things connected to the biosensors, test results of patients can be uploaded to cloud periodically, and medical workers can monitor the health patients in real‐time and take measures if necessary.^[^ ^189^ ^]^ It is conceivable that biosensors will play a crucial role in controlling the COVID‐19 pandemic shortly.

## CONFLICT OF INTEREST

The authors declare no conflict of interest.
